# Lanthanoid Biphenolates as a Rich Source of Lanthanoid‐Main Group Heterobimetallic Complexes

**DOI:** 10.1002/asia.202101328

**Published:** 2022-01-27

**Authors:** Safaa H. Ali, Angus C. G. Shephard, Jun Wang, Zhifang Guo, Murray S. Davies, Glen B. Deacon, Peter C. Junk

**Affiliations:** ^1^ College of Science & Engineering James Cook University Townsville QLD 4811 Australia; ^2^ School of Chemistry Monash University Clayton Vic 3800 Australia

**Keywords:** biphenolate ligands, coordination chemistry, heterobimetallic complexes, rare earth complexes, redox transmetallation

## Abstract

Several new trivalent dinuclear rare earth 2,2’‐methylenebis(6‐*tert*‐butyl‐4‐methylphenolate) (mbmp^2−^) complexes with the general form [Ln_2_(mbmp)_3_(thf)_n_] (Ln=Sm 1, Tb 2 (n=3), and Ho 3, Yb 4 (n=2), and a tetravalent cerium complex [Ce(mbmp)_2_(thf)_2_] (5) have been synthesised by RTP (redox transmetallation/protolysis) reactions from lanthanoid metals, Hg(C_6_F_5_)_2_ and the biphenol mbmpH_2_. These new complexes and some previously reported partially protonated rare earth biphenolate complexes [Ln(mbmp)(mbmpH)(thf)_n_] react with lithium, aluminium, potassium and zinc organometallic reagents to form lanthanoid‐main group heterobimetallic species. When reaction mixtures containing the Ln biphenolate complexes were treated with *n*‐butyllithium, both molecular ([Li(thf)_2_Ln(mbmp)_2_(thf)_n_] (Ln=La 6, Pr 7 (n=2) and Er 8, Yb 9, and Lu 10 (n=1)) and charge separated ([Li(thf)_4_][Ln(mbmp)_2_(thf)_2_] (Ln=Y 11, Sm 12, Dy 13, and Ho 14) complexes were isolated. Treatment with trimethylaluminium also led to isolation of molecular ([AlMe_2_Ln(mbmp)_2_(thf)_2_] (Ln=Pr 15, Sm 16, and Tb 17)) and ionic [La(mbmp)(thf)_5_][AlMe_2_(mbmp)] (18) complexes. One gadolinium‐potassium ([K(thf)_3_Gd(mbmp)_2_(thf)_2_] (19)), and one ytterbium‐zinc species ([ZnEtYb(mbmp)_2_(thf)] (20)) were isolated from treatment of reaction mixtures with potassium bis(trimethylsilyl)amide and diethylzinc respectively.

## Graphical abstract

**Trivalent dinuclear rare earth** 2,2′‐methylenebis(6‐tert‐butyl‐4‐methylphenolate) (mbmp^2‐^) complexes with the general form [Ln_2_(mbmp)_3_(thf)_n_] react with lithium, aluminium, potassium and zinc organometallic reagents to form lanthanoid‐main group heterobimetallic species.

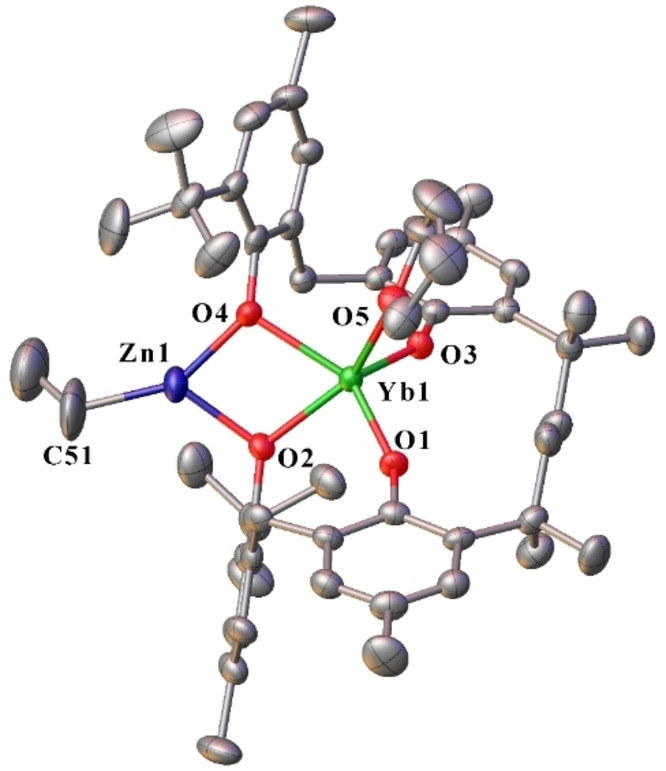

## Introduction

**Figure 1:**
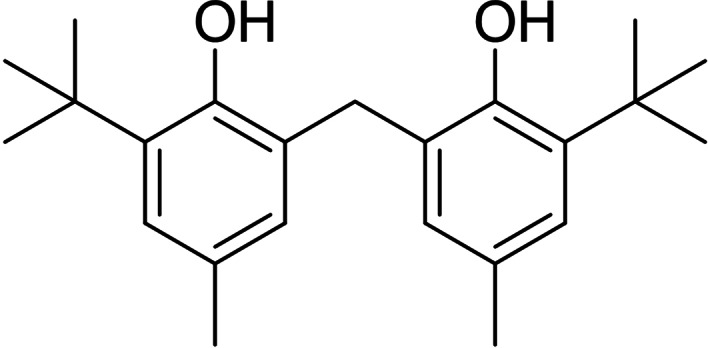
2,2’‐methylenebis(6‐*tert*‐butyl‐4‐methylphenol) (mbmpH_2_)

Lanthanoid alkoxide and aryloxide complexes have garnered significant attention in the field of coordination chemistry in recent years,^[1]^ particularly as bulky ligands for low coordinate rare earth complexes.[^1^, ^2^, ^3^] The popularity of carbon bridged biphenolate ligands has stemmed from their tunability, their ability to chelate to a metal centre reducing the potential for redistribution reactions, as well as offering a rigid framework for the metal centre, potentially affecting stereospecific transformations. Alongside this, lanthanoid biphenolate complexes have applications in sol gel methods,^[1]^ as feedstocks in MOCVD and ALD deposition of oxide layers,^[4]^ and as catalysts for ring opening polymerisation of cyclic esters.^[5]^ These biphenolate complexes have previously been synthesised by halide metathesis, or protolysis/ligand exchange reactions.[^6^, ^7^, ^8^, ^9^, ^10^, ^11^] We have previously described the synthesis of a range of lanthanoid phenolate complexes using 2,2’‐methylenebis(6‐*tert*‐butyl‐4‐methylphenol) (mbmpH_2_) (Figure 1), utilising a redox transmetallation/protolysis (RTP) approach[^12^, ^13^] from free rare earth metals, bis(pentafluorophenyl)mercury and mbmpH_2_ to yield partially deprotonated lanthanoid biphenolate complexes.^[14]^ Herein, we describe the synthesis and structures of new lanthanoid biphenolate complexes of the type [Ln_2_(mbmp)_3_(thf)_n_] by RTP reactions, and the facile ability of these complexes to form lanthanoid‐main group heterobimetallic complexes with lithium, aluminium, potassium, and zinc.

## Results and discussion

### Synthesis and Characterisation of Lanthanoid Biphenolate Complexes

The RTP reaction was used in this study to provide a simple, accessible synthetic method for a range of lanthanoid biphenolate complexes.

**Scheme 1:**
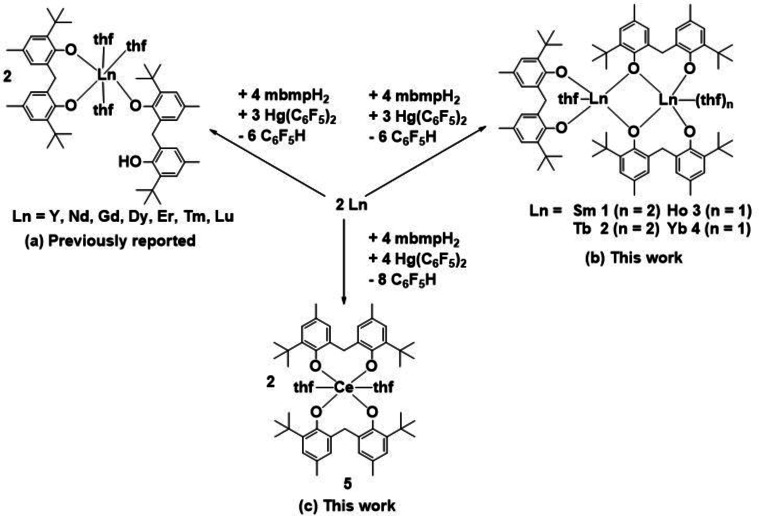
RTP reactions between mbmpH_2_, free rare earth metals and Hg(C_6_F_5_)_2_. All reactions performed in anhydrous thf at room temperature.

We have previously described a similar synthetic approach to yield mononuclear, partially deprotonated rare earth biphenolate complexes with the general form [Ln(mbmp)(mbmpH)(thf)_3_] (Ln=Y, Nd, Gd, Dy, Er Tm and Lu),^[14]^ by using an mbmpH_2_:RE metal:Hg(C_6_F_5_)_2_ ratio of 4 : 3 : 3 respectively (metal was used in excess) (Scheme 1 (a)). We now report a range of fully deprotonated trivalent dinuclear complexes, of the general form [Ln_2_(mbmp)_3_(thf)_n_] (Ln=Sm **1**, Tb **2** (n=3) and Ho **3**, Yb **4** (n=2)) (Scheme 1 (b)), and a tetravalent mononuclear cerium complex [Ce(mbmp)_2_(thf)_2_] **5** (Scheme 1 (c)). Reactions were undertaken in thf at room temperature for 3 days, with a drop of Hg metal to activate the lanthanoid metal, leading to the isolation of complexes **1**–**5** as crystals from concentrated solutions. It is noteworthy that **1**–**4** with doubly deprotonated ligands and the previously reported [Ln(mbmp)(mbmpH)(thf)_3_] complexes preferentially crystallised after similar synthesis conditions with no evidence of mixtures. The selectivity is not due to lanthanoid ion size, and perhaps solubility is a key factor.

Satisfactory microanalyses and complexometric Ln analyses were obtained for **1**–**4**, generally showing loss of solvent of crystallization from the crystal composition, as the analysis samples were dried under reduced pressure. All the Ln^3+^ ions in the complexes are paramagnetic and satisfactory ^1^H NMR spectra could not be obtained. Complex **5** was obtained only in an amount sufficient for X‐ray crystallographic identification. It is known, having been identified by crystallography, as a component of a mixture from several oxidation reactions of [Li(thf)_2_Ce(mbmp)_2_(thf)_2_].^[15]^ The infrared spectra of complexes **1**–**4** are consistent with complete deprotonation of mbmpH_2_ as the *v*(OH) bands of the biphenol (*ca*. 3600 cm^−1^ and 3390 cm^−1^) are absent.

### Structures of 1–5

X‐ray crystal structures were determined for **1**–**5** and selected bond lengths of these complexes have been summarised below with the appropriate structural figures.

**Figure 2:**
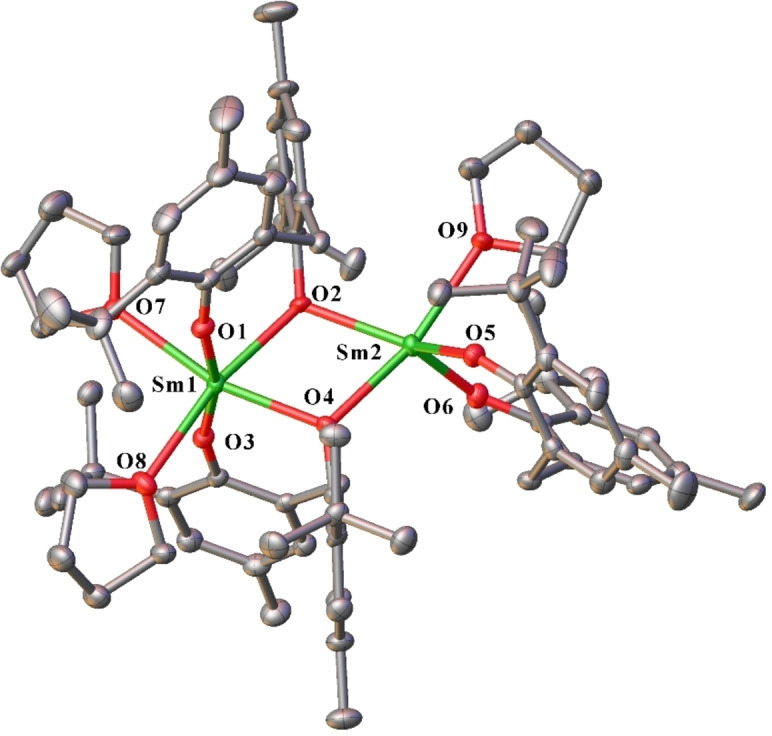
ORTEP diagram of complex **1** (also representative of **2**) showing atom‐numbering scheme for relevant atoms. Thermal ellipsoids are drawn at the 50% probability level. Hydrogen atoms are omitted for clarity. Selected bond lengths of **1** (with data for **2** in square brackets) (Å): Ln(1)‐O(1) 2.218(5) [2.192(2)], Ln(1)‐O(2) 2.358(5) [2.291(2)], Ln(1)‐O(3) 2.211(5) [2.188(2)], Ln(1)‐O(4) 2.353(5) [2.361(2)], Ln(1)‐O(7) 2.496(5) [2.425(3)], Ln(1)‐O(8) 2.504(6) [2.421(2)], Ln(2)‐O(2) 2.375(5) [2.350(2)], Ln(2)‐O(4) 2.356(5) [2.332(2)], Ln(2)‐O(5) 2.144(5) [2.119(2)], Ln(2)‐O(6) 2.145(5) [2.108(2)], Ln(2)‐O(9) 2.458(6) [2.448(2)].

Complexes **1** and **2** are isostructural with a previously reported dinuclear dysprosium complex.^[14]^ The Ln(1) atom is six‐coordinate, with distorted octahedral stereochemistry (Figure 2), and is coordinated by two bridging bidentate mbmp^2−^ ligands (O1,2; O3,4), and two *cis* thf molecules (O(7)‐Ln(1)‐O(8) 94.22(17)° (**1**) and 92.08(9)° (**2**)). The Ln(2) atom is five‐coordinate, with a distorted square pyramidal donor array. It is equatorially ligated by a single oxygen (O2,4) of two bridging mbmp^2−^ ligands, one equatorial chelating mbmp^2−^ (O5,6) and one axial thf molecule.

**Figure 3:**
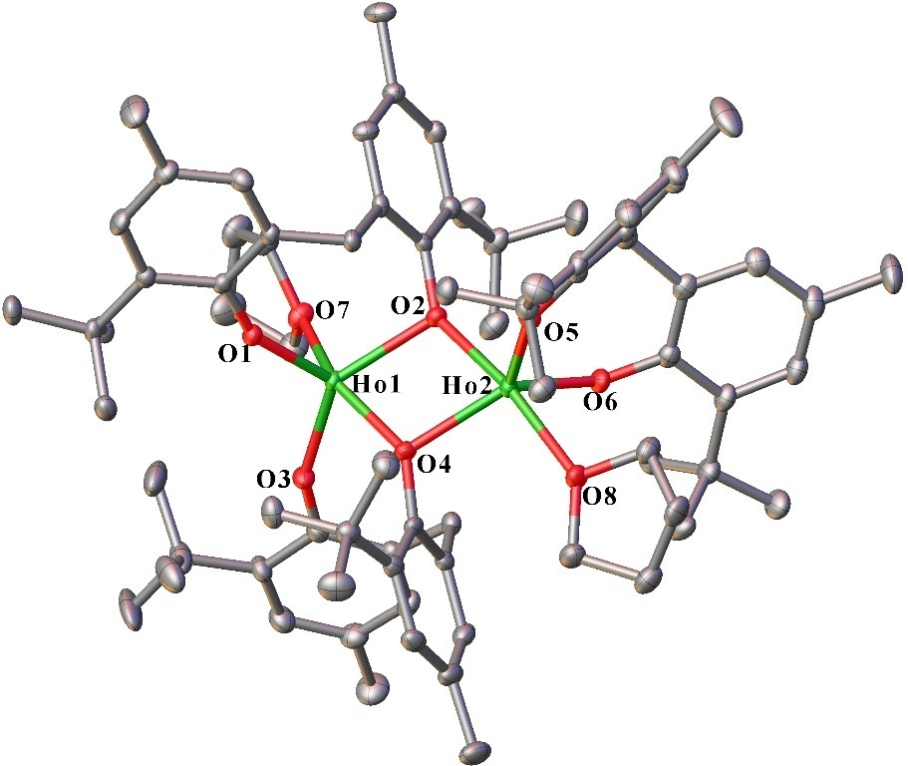
ORTEP diagram of complex **3** (also representative of **4**) showing atom‐numbering scheme for relevant atoms. Thermal ellipsoids are drawn at the 50% probability level. Hydrogen atoms are omitted for clarity. Selected bond lengths of **3** (with data for **4** in square brackets) (Å): Ln(1)‐O(1) 2.078(3) [2.062(5)], Ln(1)‐O(2) 2.259(3) [2.242(5)], Ln(1)‐O(3) 2.104(3) [2.035(5)], Ln(1)‐O(4) 2.248(3) [2.203(5)], Ln(1)‐O(7) 2.353(5) [2.317(5)], Ln(2)‐O(2) 2.270(3) [2.218(5)], Ln(2)‐O(4) 2.287(3) [2.245(5)], Ln(2)‐O(5) 2.081(3) [2.066(5)], Ln(2)‐O(6) 2.081(3) [2.052(5)], Ln(2)‐O(8) 2.382(3) [2.365(5)].

Complexes **3** and **4** are isostructural, and the composition varies from complexes **1** and **2** by one less coordinated thf molecule. Both metal atoms have a distorted square pyramidal stereochemistry (Figure 3). Ln(1) is coordinated by two bridging, bidentate mbmp^2−^ ligands, with O(1) and O(3) terminal, and O(2) and O(4) bridging to Ln(2), and a thf donor. The other metal atom, Ln(2), is coordinated by the bridging oxygens, a terminal, chelating mbmp^2−^ ((O(5) and O(6)), and a thf molecule.

**Figure 4:**
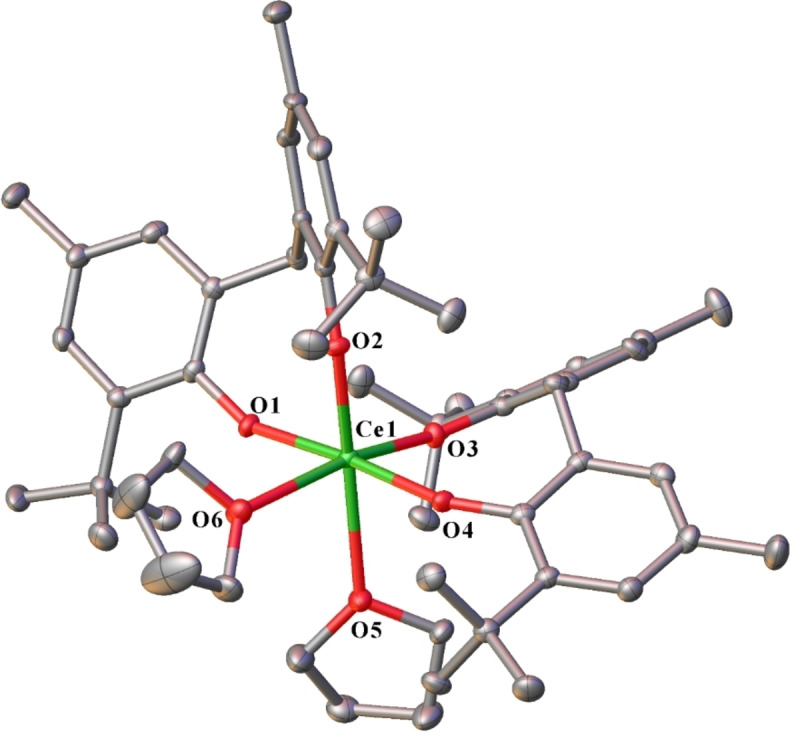
ORTEP diagram of complex **5** showing atom‐numbering scheme for relevant atoms. Thermal ellipsoids are drawn at the 50% probability level. Hydrogen atoms are omitted for clarity. Selected bond lengths (Å): Ce(1)‐O(1) 2.153(3), Ce(1)‐O(2) 2.130(3), Ce(1)‐O(3) 2.113(3), Ce(1)‐O(4) 2.147(3), Ce(1)‐O(5) 2.507(4), Ce(1)‐O(6) 2.522(4).

Complex **5** is comprised of a six‐coordinate cerium centre, with a distorted octahedral donor array (Figure 4). The cerium atom is coordinated by two chelating mbmp^2−^ ligands, and two *cis* thf molecules. The metrical parameters agree with those reported.^[15]^

### Reactions to form Heterobimetallics

Lanthanoid biphenolate heterobimetallic chemistry is still limited and few complexes have been reported. We previously reported the synthesis of an yttrium‐aluminium bimetallic complex from treating the partially protonated yttrium biphenolate complex [Y(mbmp)(mbmpH)(thf)_3_] with trimethylaluminium,^[14]^ and the Yb analogue was previously prepared by the same method.^[7]^ We have since expanded this approach to synthesise a range of new rare earth heterobimetallics with lithium, aluminium, potassium, and zinc. An Sm−Al mbmp tetramethylaluminate complex has previously been prepared by protolysis of [Sm(AlMe_4_)_3_] with mbmpH_2_ and was treated with azobenzene to give a dimethylaluminum derivative.^[16]^ Other molecular rare earth‐potassium heterobimetallic biphenolate complexes, namely of Sm and Yb have previously been synthesised by metathesis reactions of mbmpK_2_ with the corresponding rare earth chloride.^[11]^

### Reactions with n–butyllithium

**Scheme 2:**
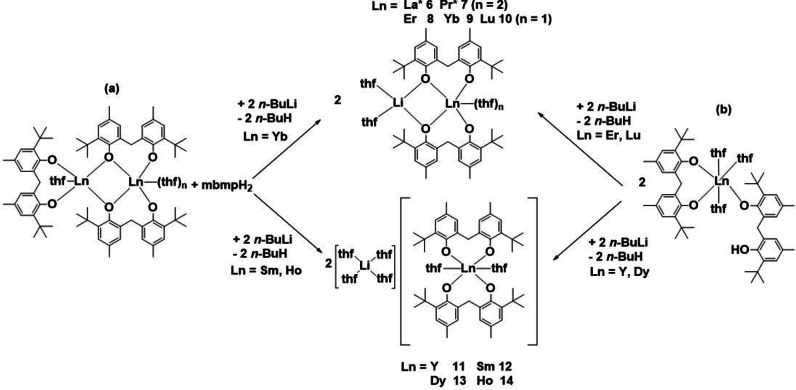
Reactions of *in situ* formed (a) dinuclear and (b) mononuclear partially deprotonated rare earth biphenolate complexes with *n*‐butyllithium to form heterobimetallics. All reactions were performed in anhydrous thf at room temperature.

When RTP reaction mixtures (a) for the formation of **1**, **3**, and **4** or (b) for the formation of [Ln(mbmp)(mbmpH)thf)_3_] (Ln=Dy, Y, Er, Lu) or (c) for the formation of putative La and Pr analogues which so far have not been crystallised, were treated with *n*‐butyllithium (Ln : Li=1 : 1), heterobimetallic complexes **6**–**14** were successfully obtained (Scheme 2 (a)‐(c) respectively). Irrespective of the synthetic route, the resulting heterobimetallic was either molecular (complexes **6**–**10**), or a discrete cation‐anion pair (complexes **11**–**14**). Elemental analyses of the complexes were determined after drying under reduced pressure, and therefore some exhibited loss of lattice solvent and in some cases coordinated solvent from the crystal composition as determined by X‐ray crystallography. Thus, microanalyses of **6** and **7**, which have no lattice solvent, corresponded to the crystal composition. Complex **8** exhibited loss of two lattice C_6_D_6_ and two coordinated thf, **10** loss of three C_6_D_6_ lattice solvent**, 11** loss of one lattice thf and two coordinated thf, **12** and **13** exhibited loss of 0.5 lattice thf, and **14** exhibited loss of one lattice thf and four coordinated thf molecules. These results were supported by complexometric titration to determine the % rare earth metal. Crystals of complex **9** were isolated, but only in low yields, hence only an X‐ray crystal structure could be obtained.

IR spectra of complexes **6**–**14** are consistent with complete deprotonation of the mbmpH_2_ starting material (no *v*(OH) absorption). Of these complexes only the ^1^H NMR spectra of **6** and **10** were able to be recorded and interpreted, and both confirm biphenolate:thf ratios observed in the X‐ray crystal structures. The molecular Li/Ln complexes show the bridging CH_2_ resonance split into an apparent AB doublet (slightly broadened in the case of **10**).

**Figure 5:**
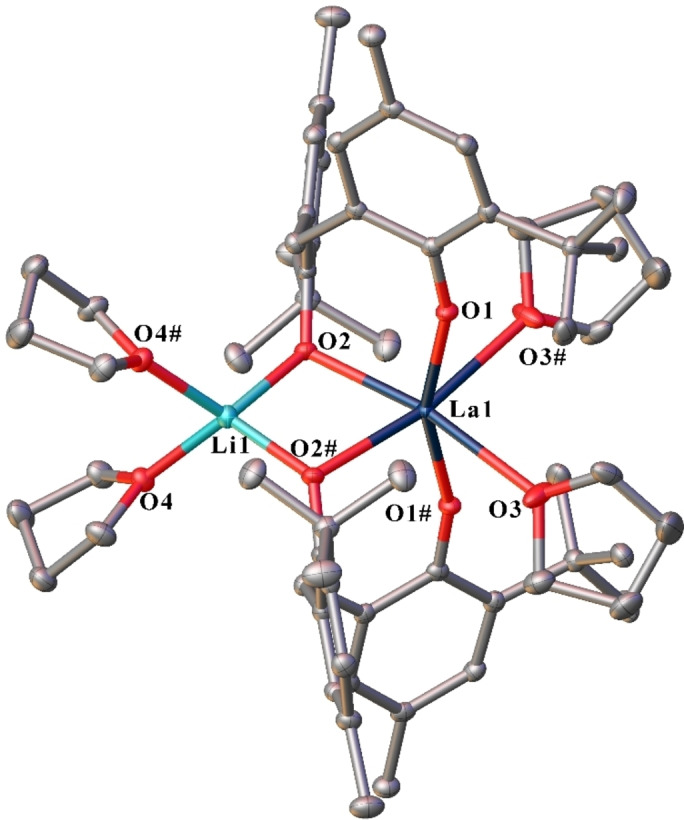
ORTEP diagram of complex **6** (also representative of **7**) showing atom‐numbering scheme for relevant atoms. Thermal ellipsoids are drawn at the 50% probability level. Hydrogen atoms are omitted for clarity. Selected bond lengths of **6** (with data for **7** in square brackets) (Å): Ln(1)‐O(1) 2.3408(16) [2.267(5)], Ln(1)‐O(2) 2.3948(15) [2.366(5)], Ln(1)‐O(3) 2.5989(18) [2.581(5)], Li(1)‐O(2) 1.969(4) [1.887(15)], Li(1)‐O(4) 2.020(4) [1.99(2)].

Complexes **6** and **7** are isomorphous (Table S1) molecular compounds and are comprised of a six‐coordinate, distorted octahedral Ln atom, bridged by phenolate oxygen atoms to a four‐coordinate, distorted tetrahedral Li atom (Figure 5). The Ln atom is bound by two mbmp^2−^ ligands (O1,2 and symmetry equivalent), with one oxygen of each bound solely to the Ln atom in the axial positions, and the other oxygen of each bridging between the Ln and the Li atoms, and two *cis* thf molecules in the equatorial sites. The Li atom is bound by two bridging mbmp^2−^ oxygens, and two thf molecules. The bridging oxygens of the two mbmp^2−^ ligands have considerably longer Ln−O bond lengths than their non‐bridging counterparts.

**Figure 6:**
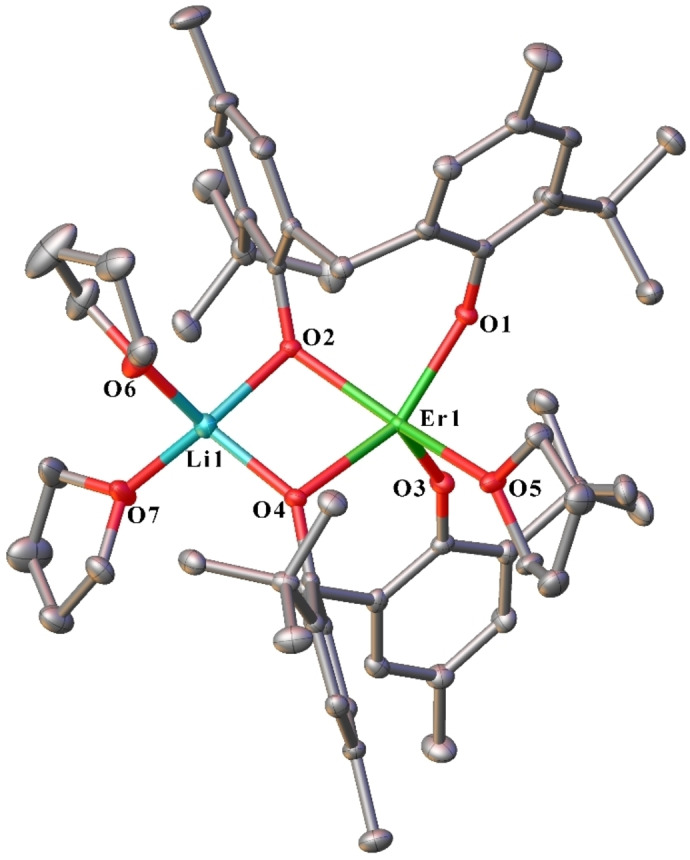
ORTEP diagram of complex **8** (also representative of **9** and **10**) showing atom‐numbering scheme for relevant atoms. Thermal ellipsoids are drawn at the 50% probability level. Hydrogen atoms are omitted for clarity. Selected bond lengths of **8** (with data for **9** and **10** respectively in square brackets) (Å): Ln(1)‐O(1) 2.1097(16) [2.070(19), 2.0724(19)], Ln(1)‐O(2) 2.1711(18) [2.2068(18), 2.1502(17)], Ln(1)‐O(3) 2.0763(17) [2.080(2), 2.0475(18)], Ln(1)‐O(4) 2.2434(15) [2.1552(18), 2.1613(17)], Li(1)‐O(2) 2.004(5) [1.943(5), 1.993(5)], Li(1)‐O(4) 1.957(4) [2.031(5), 1.984(5)], Ln(1)‐O(5) 2.3598(18) [2.3454(19), 2.3213(19)], Li(1)‐O(6) 1.969(5) [2.031(5), 1.966(5)], Li(1)‐O(7) 1.993(4) [1.974(5), 2.009(4)].

Complexes **8**–**10** are isostructural molecular compounds and are comprised of a five‐coordinate, distorted square pyramidal Ln atom, linked to a four‐coordinate, distorted tetrahedral Li atom (Figure 6). Similar to complexes **6** and **7**, the Ln centre is coordinated by two mbmp^2−^ ligands (O1,2 and O3,4), each with one terminal oxygen, and one oxygen bridging the Ln and Li atoms, and one thf. The two bridging mbmp^2−^ oxygens, and two molecules of thf ligate the Li atom. Again, the bridging Ln−O bond lengths are considerably longer than their non‐bridging counterparts. A previously prepared Yb−Li analogue^[7]^ is isostructural and differs only in the associated lattice solvent (1 PhMe and 0.5thf)^[7]^ vs hexane (Table S1).

**Figure 7:**
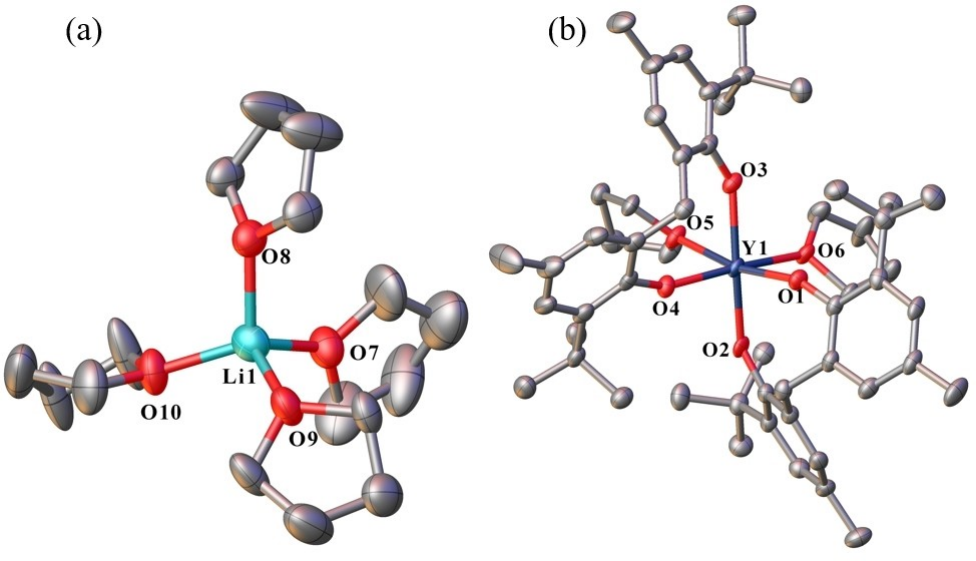
ORTEP diagram of complex **11** ((a)=[Li(thf)_4_]^+^ and (b)=[Ln(mbmp)_2_(thf)_2_]^−^) (also representative of **12–14**) showing atom‐numbering scheme for relevant atoms. Thermal ellipsoids are drawn at the 50% probability level. Hydrogen atoms are omitted for clarity. Selected bond lengths have been summarised in Table 1. Selected bond lengths of **11** (with data for **12–14** respectively in square brackets)(Å): Ln(1)‐O(1) 2.121(3) [2.2595(17), 2.207(2), 2.201(2)], Ln(1)‐O(2) 2.183(4) [2.176(2), 2.144(3), 2.125(3)], Ln(1)‐O(3) 2.207(4) [2.2137(17), 2.175(2), 2.160(2)], Ln(1)‐O(4) 2.131(4) [2.2718(17), 2.213(2), 2.213(2)], Ln(1)‐O(5) 2.438(3) [2.513(2), 2.453(3), 2.439(3)], Ln(1)‐O(6) 2.438(4) [2.523(2), 2.459(2), 2.444(2)].

Complexes **11**–**14** are isostructural ionic compounds, with a six‐coordinate, octahedral Ln atom in the anion, and a four‐coordinate, tetrahedral Li atom in the cation (Figure 7). The Ln centre is bound by two fully deprotonated mbmp^2−^ ligands, and two *cis* thf molecules (e. g. O(5)‐Sm(1)‐O(6)=82.46(7)°) in the equatorial positions. The Ln−O bonds of the mbmp^2−^ ligands are considerably longer in the axial positions. The [Li(thf)_4_]^+^ cation is widely reported in the literature, with 595 structural studies.^[17]^ Similar ionic rare earth‐lithium biphenolate/amide heterobimetallics with the general form [Li(thf)_4_][Ln(mbmp)(N(SiCH_3_)_3_)_2_] (Ln=Nd and Yb) have previously been synthesised by treatment of chloride bridged rare earth biphenolate complexes with lithium bis(trimethylsilyl)amide,^[18]^ where the [Li(thf)_4_]^+^ cation shows slightly longer Li‐O_(thf)_ bond lengths (average Li‐O_(thf)_=1.908 Å) than complexes **11**–**14** (average Li‐O_(thf)_=1.845 Å). The difference in bond length could be attributed to elevated temperatures during data collection for the [Li(thf)_4_][Ln(mbmp)(N(SiCH_3_)_3_)_2_] compounds (193 K vs 100 K for **11**–**14**).

Just as there was no obvious correlation between which Ln elements form [Ln(mbmp)(mbmpH)(thf)_n_] and which give [Ln_2_(mbmp)_3_(thf)_n_] complexes in RTP reactions, the factors deciding which Ln metals give molecular Li/Ln bimetallics (La, Pr, Y, Er, Yb, Lu) and which give charge separated species (Sm, Dy, Ho) are not clear. Whilst the latter appear associated with mid‐sized Ln^3+^, a break between Ho and Y/Er is surprising. The division cannot be correlated with the two different classes of reagent, as some from each class fall into each class of heterobimetallics. Moreover, the molecular/ charge separated division for Li/Ln bimetallics does not relate to the division within Al/Ln bimetallics (below) where La and Pr fall into different classes and Sm now gives a molecular Al/Sm species. It may be that solubilities and crystallization conditions decide the outcome.

### Reactions with trimethylaluminium

**Scheme 3:**
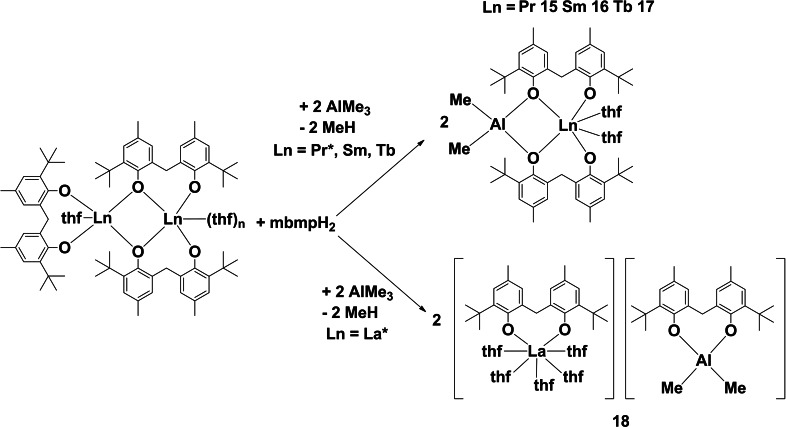
Reactions of dinuclear rare earth complexes with trimethylaluminium to yield heterobimetallics. *The precursors of complexes **15** and **18** could not be isolated. All reactions were performed in anhydrous thf at room temperature.

Several rare earth‐aluminium heterobimetallics were synthesised in a similar fashion to the rare earth‐lithium heterobimetallics: namely by treatment of the RTP reaction mixtures containing the dinuclear complexes [Ln_2_(mbmp)_3_(thf)_n_] (from reaction of mbmpH_2_ with La, Pr, Sm and Tb metals and Hg(C_6_F_5)2_) with trimethylaluminium (Al: Ln=1 : 1; Scheme 3). This method yielded either molecular rare earth‐aluminium heterobimetallics with the general form [AlMe_2_Ln(mbmp)_2_(thf)] (Ln=Pr (**15**), Sm (**16**), and Tb (**17**)), or a discrete cation‐anion pair in [AlMe_2_(mbmp)][La(mbmp)(thf)_4_] (**18**) (Scheme 3).

Elemental analyses were collected after drying under reduced pressure. Complex **15** exhibited loss of two lattice C_6_D_6_, **16** exhibited loss of one and half lattice C_6_D_6_ and one coordinated thf, **17** loss of two lattice C_6_D_6_, and **18** exhibited loss of three coordinated thf molecules. These results were supported by complexometric titration to determine the % Ln. In the IR spectra of complexes **15**–**18** no *v*(OH) absorptions were observed signifying total deprotonation of the mbmpH_2_ ligand. The ^1^H NMR spectrum of paramagnetic **17** was able to be collected and interpreted, confirming biphenolate:thf:Me_(Al)_ ratios. Complex **17** also shows the splitting of the bridging CH_2_ resonance observed for the Li/Ln heterobimetallics, but this feature is shifted to lower energies, whereby one of the signals is slightly masked by the thf resonance.

**Figure 8:**
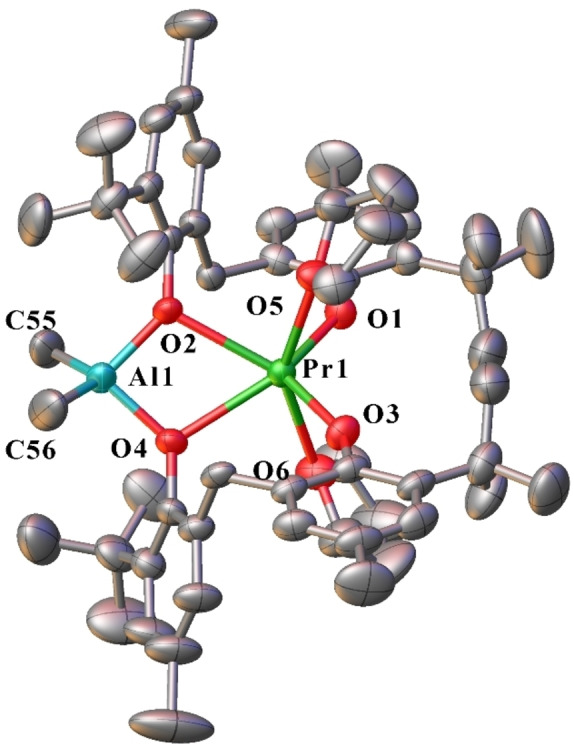
ORTEP diagram of complex **15** (also representative of **16** and **17**) showing atom‐numbering scheme for relevant atoms. Thermal ellipsoids are drawn at the 50% probability level. Hydrogen atoms are omitted for clarity. Selected bond lengths of **15** (with data for **16** and **17** respectively in square brackets) (Å): Ln(1)‐O(1) 2.119(4) [2.162(3), 2.188(6)], Ln(1)‐O(2) 2.507(4) [2.447(3), 2.515(6)], Ln(1)‐O(3)2.171(4) [2.143(3), 2.493(6)], Ln(1)‐O(4)2.497(4) [2.447(3), 2.162(6)], Ln(1)‐O(5) 2.533(4) [2.466(3), 2.527(7)], Ln(1)‐O(6) 2.561(4) [2.475(3), 2.557(6)], Al(1)‐O(2)1.817(4) [1.829(3), 1.835(6)], Al(1)‐O(4)1.830(4) [1.833(3), 1.838(6)], Al(1)‐C(55) 1.982(8) [1.969(5), 1.976(12)], Al(1)‐C(56) 1.927(9) [1.970(5), 1.969(11)].

Complexes **15**–**17** are isostructural molecular compounds, comprised of a six‐coordinate, distorted trigonal prismatic Ln atom, linked to a four‐coordinate, distorted tetrahedral Al atom (Figure 8). Ln(1) is coordinated by two bridging mbmp^2−^ ligands, and two *transoid* thf molecules (O(5)‐Ln(1)‐O(6)=149.45(13)° (**15**), 148.71(10)° (**16**), 150.1(2)° (**17**)). One oxygen of each mbmp^2−^ ligand is coordinated solely to the Ln, whilst the other is bridging between the Ln and Al atoms. The aluminium atom is coordinated by the two bridging mbmp^2−^ oxygens, and two methyl groups. Again, the Ln−O bond lengths of the bridging oxygens are significantly longer than their non‐bridging counterparts. Analogous rare earth‐aluminium biphenolate bimetallics with the general form [AlMe_2_Ln(mbmp)_2_(thf)_2_] (Ln=Y,^[14]^ and Sm^[16]^) have been reported, the former from reaction of an isolated [Y(mbmp)(mbmpH)(thf)_3_] complex and there are analogous lanthanum‐ and cerium‐aluminium based anilido complexes.^[19]^

**Figure 9:**
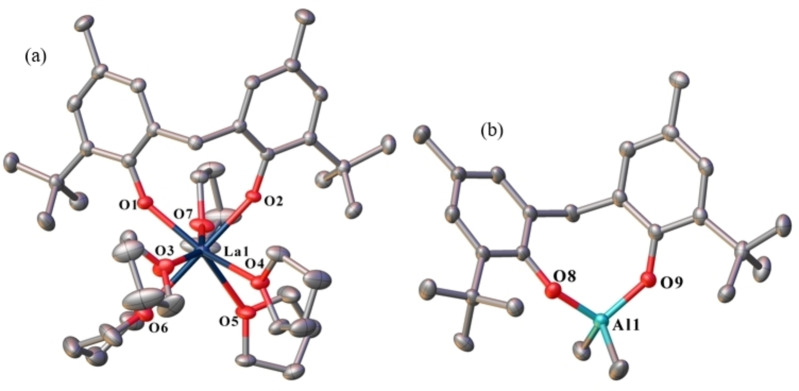
ORTEP diagram of complex **18** ((a)=[La(mbmp)(thf)_5_]^+^ and (b)=[AlMe_2_(mbmp)]^−^) showing atom‐numbering scheme for relevant atoms. Thermal ellipsoids are drawn at the 50% probability level. Hydrogen atoms are omitted for clarity. Selected bond lengths (Å): La(1)‐O(1) 2.2282(19), La(1)‐O(2) 2.2460(19), Al(1)‐O(8) 1.765(2), Al(1)‐O(9) 1.785(2), La(1)‐O(3) 2.595(2), La(1)‐O(4) 2.609(2), La(1)‐O(5) 2.598(2), La(1)‐O(6) 2.638(3), La(1)‐O(7) 2.572(2).

Complex **18** is an ionic compound, comprised of a seven‐coordinate, distorted pentagonal bipyramidal lanthanum cation and a four‐coordinate, distorted tetrahedral aluminium anion (Figure 9). The lanthanum atom is ligated by one mbmp^2−^ ligand, one oxygen in an axial, and the other in an equatorial position (O(1)‐La(1)‐O(2)=92.93(7)°) and five thf molecules, one in an axial, and four in equatorial positions. The aluminium atom is ligated by two methyl groups, and one chelating mbmp^2−^ ligand.

### A reaction with potassium bis(trimethylsilyl)amide

**Scheme 4:**
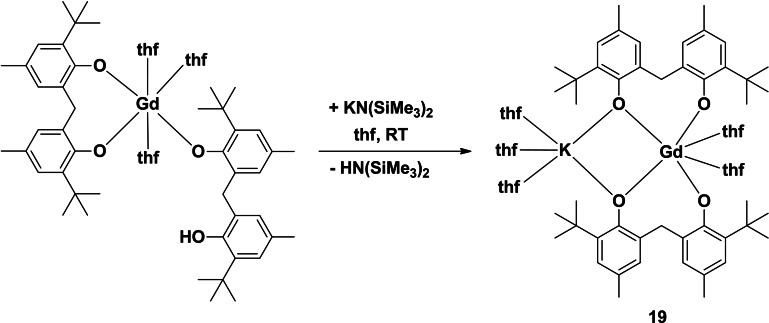
Reaction of a partially protonated, mononuclear Gd complex with potassium bis(trimethylsilyl)amide to yield a Gd−K bimetallic complex (**19**).

One rare earth‐potassium heterobimetallic complex was isolated, which was from the reaction of [Gd(mbmp)(mbmpH)(thf)_3_]^[14]^ with one equivalent of KN(SiMe_3_)_2_, yielding [K(thf)_3_Gd(mbmp)_2_(thf)_2_] (**19**) (Scheme 4). The IR spectrum of **19** shows complete deprotonation of the mbmpH_2_ ligand by the absence of a *v*(OH) band. An interpretable ^1^H NMR spectrum could not be collected due to the paramagnetic nature of Gd^3+^. The elemental analysis of **19** after drying under vacuum showed loss of one half of a lattice thf molecule, and this was supported by complexometric titration to determine % Gd.

**Figure 10:**
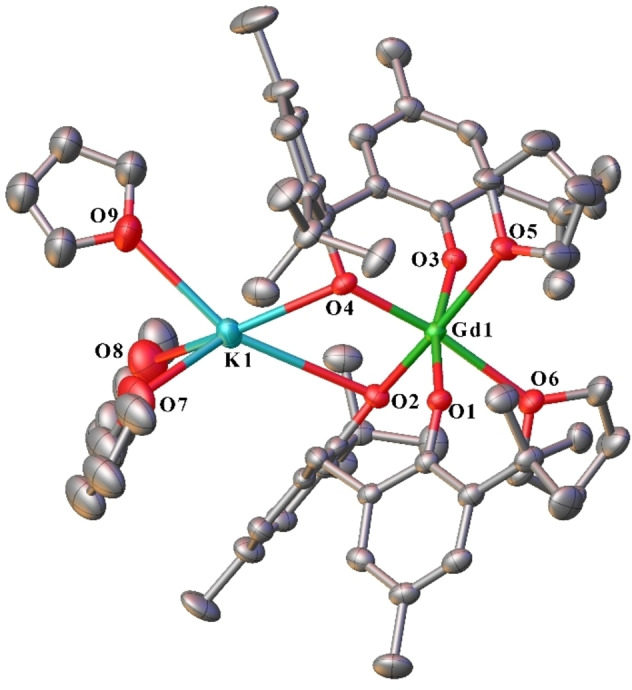
ORTEP diagram of complex **19** showing atom‐numbering scheme for relevant atoms. Thermal ellipsoids are drawn at the 50% probability level. Hydrogen atoms are omitted for clarity. Selected bond lengths (Å): Gd(1)‐O(1) 2.228(5), Gd(1)‐O(2) 2.170(5), Gd(1)‐O(3) 2.210(5), Gd(1)‐O(4) 2.233(5), Gd(1)‐O(5) 2.572(4), Gd(1)‐O(6) 2.515(4), K(1)‐O(2) 2.787(5), K(1)‐O(4) 2.787(5), K(1)‐O(7) 2.695(14), K(1)‐O(8) 2.724(18), K(1)‐O(9) 2.916(16).

**Figure 11:**
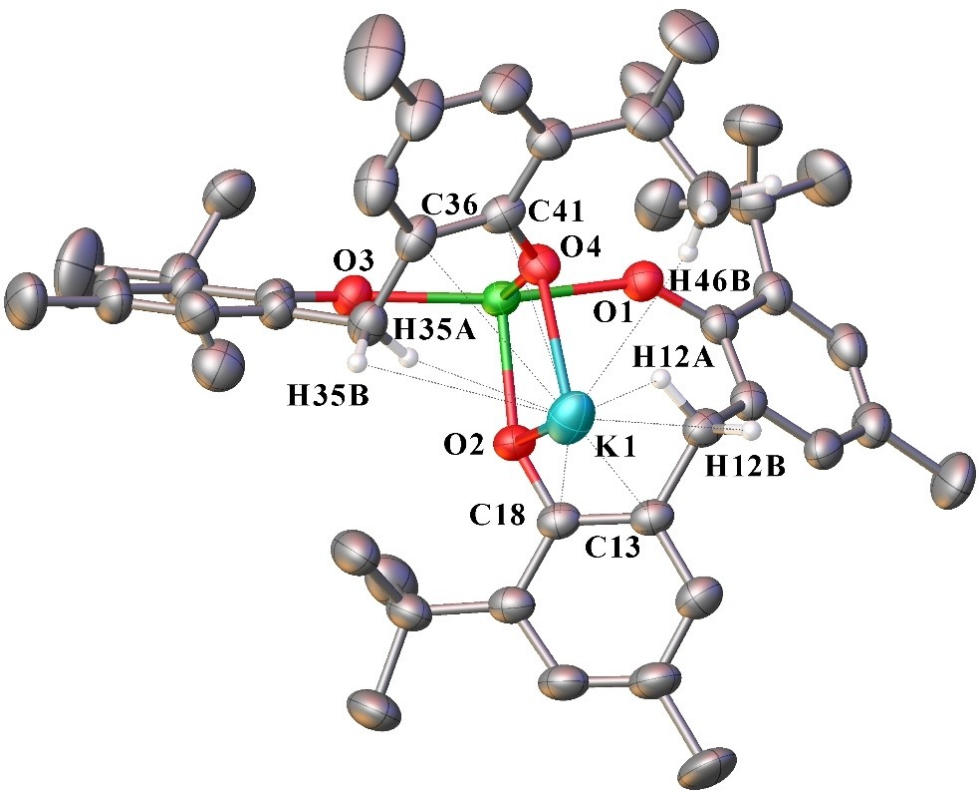
Agostic interactions of 11 showing atom‐numbering scheme for atoms involved in Table 1. Coordinated thf and hydrogen atoms not involved in the coordination sphere of K are omitted for clarity.

Complex **19** consists of a six‐coordinate, octahedral gadolinium atom linked to a five‐coordinate, distorted square pyramidal potassium atom (Figure 10). The gadolinium atom is ligated by two bridging mbmp^2−^ ligands, and two *cis* equatorial thf molecules (O(5)‐Gd(1)‐O(6)=94.81(16)°). One oxygen of each mbmp^2−^ ligand is coordinated solely to the Gd, whilst the other is bridging between the Gd and K atoms. The potassium atom is ligated by three thf molecules, and two bridging mbmp^2−^ oxygens. Since this is a low coordination number for the large potassium ion, we have investigated adjacent carbon and hydrogen atoms for the possibility that they ae are contributing electron density to the potassium atom (Table 1 and Figure 11). From consideration of K−C bond lengths in K(η^6^ –arene) ^+^ complexes, K−C distances of <3.5 Å can be considered an interaction^[20]^ and from a range of other structures, K−H may be interacting.[^21^, ^22^, ^23^, ^24^] On this basis an *ipso* and an *ortho* carbon from each mbmp^2−^ ligand, can be considered to interact with potassium, but the conclusion has to be tempered by the role of the binding of the phenolate oxygen in bringing the carbon atoms near K. Likewise, one C−H of each methylene could make an agostic interaction. On the other hand, the second methylene C−H and the closest H of the *t*Bu group are too distant to be considered an interaction. Overall, there are a collection of nearby C and H atoms that can provide additional electron density to K whilst some of the groups also provide steric stabilisation.

**Table 1:** Summary of agostic K−C and K−H interactions

Bond	Bond distance [Å]	K−C of accompanying carbon [Å]
K(1)‐C(18)	3.369(5)	–
K(1)‐C(13)	3.324(5)	–
K(1)‐C(41)	3.211(5)	–
K(1)‐C(36)	3.480(6)	–
K(1)‐H(46B)	3.0534(18)	3.800(8)
K(1)‐H(12 A)	2.8893(17)	3.382(5)
K(1)‐H(12B)	3.3630(17)	3.382(5)
K(1)‐H(35 A)	2.8039(17)	3.339(6)
K(1)‐H(35B)	3.2488(16)	3.339(6)

As observed previously, the Ln−O bond lengths of the bridging oxygens are considerably longer than their non‐bridging counterparts.

### A reaction with diethylzinc

**Scheme 5:**
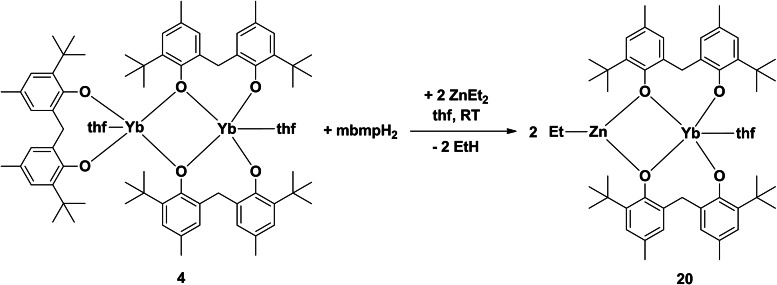
Reaction of a dinuclear Yb complex (**4**) with diethylzinc yielding an Yb−Zn bimetallic complex (**20**).

Previous attempts of synthesising rare earth‐zinc biphenolate heterobimetallic complexes, by treatment of partially protonated rare earth biphenolates ([Ln(mbmp)(mbmpH)(thf)_2_] (Ln=Y, and Yb)) with diethylzinc resulted in redistribution, yielding a zinc biphenolate complex, [Zn(mbmp)_2_(thf)]_2_ instead of the targeted heterobimetallic.^[7]^ However, when complex **4** was treated with two equivalents of diethylzinc in the presence of one equivalent of mbmpH_2_, [ZnEtYb(mbmp)_2_(thf)] (**20**) was isolated (Scheme 5). The IR spectrum of **20** displayed no *v*(OH) absorption, confirming complete deprotonation of mbmpH_2_. Yb^3+^ is paramagnetic in nature and no interpretable ^1^H NMR spectrum of **20** could be collected. The elemental analysis after drying under vacuum showed loss of one and a half lattice C_6_D_6_ molecules (out of two in the single crystal composition).

**Figure 12:**
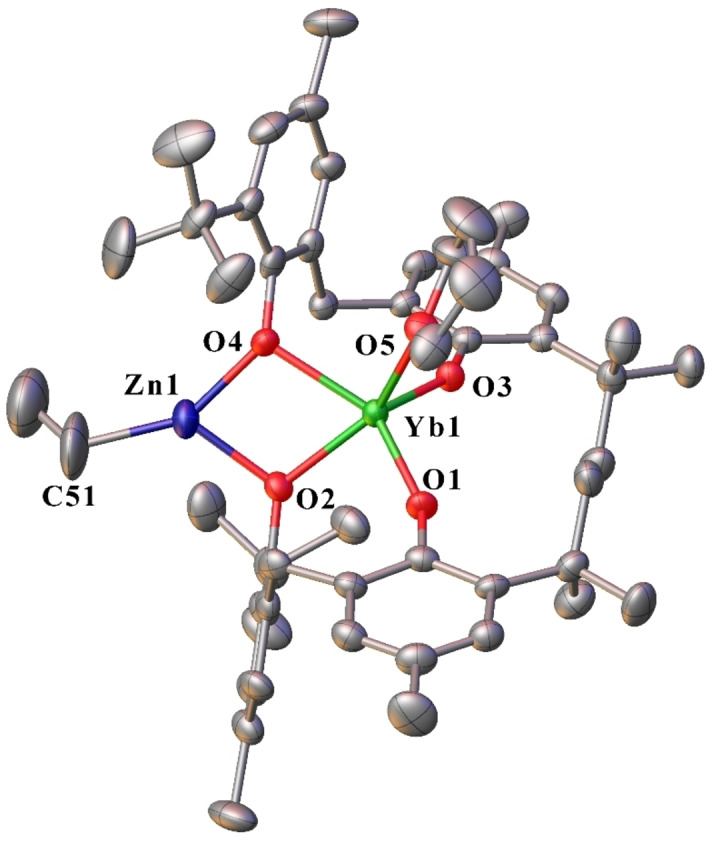
– ORTEP diagram of complex **20** showing atom‐numbering scheme for relevant atoms. Thermal ellipsoids are drawn at the 50% probability level. Hydrogen atoms are omitted for clarity. Selected bond lengths (Å): Yb(1)‐O(1) 2.049(5), Yb(1)‐O(2) 2.215(6), Yb(1)‐O(3) 2.031(4), Yb(1)‐O(4) 2.246(7), Zn(1)‐O(2) 1.976(6), Zn(1)‐O(4) 1.961(8), Yb(1)‐O(5) 2.302(6), Zn(1)‐C(51) 1.962(13)

Complex **20** is made up of a five‐coordinate ytterbium atom in a distorted square pyramidal geometry, and a three‐coordinate zinc atom in a distorted trigonal planar geometry (Figure 12). The ytterbium atom is bound by two bridging mbmp^2−^ ligands, and one thf molecule. One oxygen of each mbmp^2−^ ligands is coordinated solely to the Yb, whilst the other is bridging between the Yb and Zn atoms. The latter also has an ethyl group bound to it, giving Zn a coordination number of three. The bridging Zn−O bond lengths are similar to those of [Zn(mbmp)(thf)]_2_.^[7]^

## Conclusions

A variety of dinuclear rare earth biphenolate complexes of the general form [Ln_2_(mbmp)_3_(thf)_n_] (Ln=Sm **1**, Tb **2** (n=2) and Ho **3**, Yb **4** n=3), alongside one previously reported cerium(IV) complex [Ce(mbmp)_2_(thf)_2_] (**5**), have been synthesised by RTP reactions between free Ln metals, Hg(C_6_F_5_)_2,_ and mbmpH_2_. These dinuclear complexes, as well as some known partially protonated [Ln(mbmp)(mbmpH)(thf)_n_] complexes, generated in situ, were treated with various organometallic bases (lithium, aluminium, potassium and zinc reagents) to yield a range of heterobimetallic complexes. Use of in situ generated complexes greatly simplifies the bimetallic synthesis. Two types of rare earth–lithium bimetallic complexes were obtained, namely molecular compounds with the general form [Li(thf)_2_Ln(mbmp)_2_(thf)_n_] (Ln=La **6**, Pr **7** (n=2) and Er **8**, Yb **9**, and Lu **10** (n=1)) or ionic species with the general form [Li(thf)_4_][Ln(mbmp)_2_(thf)_2_] (Ln=Y **11**, Sm **12**, Dy **13**, and Ho **14**). Similarly, treatment with trimethylaluminium led to both molecular compounds with the general form [AlMe_2_Ln(mbmp)_2_(thf)_2_] (Ln=Pr **15**, Sm **16**, and Tb **17**), and an ionic species in the case of lanthanum, [La(mbmp)(thf)_5_][AlMe_2_(mbmp)] **18**. One each of potassium and zinc heterobimetallic species, namely [K(thf)_3_Gd(mbmp)_2_(thf)_2_] **19** and [ZnEtYb(mbmp)_2_(thf)] **20** were also isolated from reactions with potassium bis(trimethylsilyl)amide and diethylzinc respectively. The structures of the molecular heterobimetallics feature mbmp^2−^ ligands bridging through one of the phenolate oxygens with the other bound solely to the lanthanoid metal.

## Experimental

### Materials and General Procedures

All manipulations were performed under nitrogen, using standard Schlenk and drybox techniques. Solvents (thf and toluene) were distilled from sodium benzophenone before use. 2,2’‐Methylene‐bis(6‐*tert*‐butyl‐4‐methylphenol), *n*‐butyllithium, trimethylaluminium, and potassium bis(trimethylsilyl)amide, and diethylzinc were commercially available, and used without further purification. Bis(pentafluorophenyl)mercury was prepared by the literature method.^[25]^ Metal analyses were determined by Na_2_H_2_edta titration with a Xylenol Orange indicator and hexamethylenetetramine buffer, after decomposition of complexes with dilute HCl. For the heterobimetallic complexes, aluminium was masked in this process by addition of 5% sulfosalicylic acid solution.^[26]^ Infrared spectra (4000–400 cm^−1^) were obtained as Nujol mulls between NaCl plates with a Nicolet**‐**Nexus FT**‐**IR spectrometer. ^1^H**‐**NMR spectra were recorded on a Bruker 400 MHz spectrometer. The chemical shifts were referenced to residual solvent peaks. Crystal data and refinement details are given in **Table S1**. CCDC 2124516‐2124535 for compound **1**–**20**, contain the supplementary crystallographic data for this paper. These data can be obtained free of charge from The Cambridge Crystallographic Data Centre via www.ccdc.cam.ac.uk/data_request/cif.

### Syntheses

#### [Sm_2_(mbmp)_3_(thf)_3_] ⋅ 6 thf (1)

A Schlenk flask equipped with a magnetic stirrer bar was charged with mbmpH_2_ (1.36 g; 4.00 mmol), Hg(C_6_F_5_)_2_ (1.60 g; 3.00 mmol), one drop of Hg metal (to form a reactive lanthanoid‐mercury amalgam) and excess samarium filings (0.45 g; 3.0 mmol). Anhydrous thf (∼20 mL) was added by cannula, and the reaction mixture stirred at room temperature for 3 days. Excess samarium metal and mercury were allowed to settle before isolating the supernatant liquid by a filtration cannula. The resulting filtrate was concentrated under reduced pressure to ∼5 mL and allowed to stand at room temperature to crystallise, yielding colourless crystals (0.40 g, 26%). M. p. 218–220 °C; *Anal*. Calc. for C_81_H_114_O_9_Sm_2_ (1532.49 g.mol^−1^ after loss of six lattice thf): C 63.48, H 7.50, Sm 19.62. Found: C 63.19, H 7.11, Sm 19.14%. IR (Nujol, cm^−1^): 2058 w, 1750 m, 1249 s, 1138 s, 1060 m, 1011 m, 917 s, 863 s, 814 s, 794 s, 724 m, 670 s.

#### [Tb_2_(mbmp)_3_(thf)_3_] ⋅ 2 C_6_D_6_ (2)

Synthesised as per **1** but with terbium filings (0.47 g, 3.00 mmol) in place of samarium. Colourless crystals grew overnight from C_6_D_6_ (0.32 g, 21%) M. p. 243–245 °C; *Anal*. Calc. for C_81_H_114_O_9_Tb_2_ (1549.62 g.mol^−1^ after loss of two lattice C_6_D_6_): C 62.78, H 7.42, Tb 20.51. Found: C 62.25, H 7.19, Tb 20.12%. IR (Nujol, cm^−1^): 1738 w, 1565 m, 1528 w, 1463 s, 1376 s, 1266 s, 1204 m, 1171 w, 1138 m, 1073 m, 1007 s, 913 s, 859 s, 818 s, 789 m, 724 w.

#### [Ho_2_(mbmp)_3_(thf)_2_] ⋅ 3 C_6_D_6_ (3)

Synthesised as per **1** but with holmium filings (0.49 g, 3.00 mmol) in place of samarium. Colourless crystals grew overnight from C_6_D_6_ (0.35 g, 24%). M. p. 243–245 °C; *Anal*. Calc. for C_77_H_106_O_8_Ho_2_ (1489.52 g.mol^−1^ after loss of three lattice C_6_D_6_): C 62.09, H 7.17, Ho 22.15. Found: C 61.85, H 7.08, Ho 22.06%. IR (Nujol, cm^−1^): 2284 w, 1936 m, 1854 m, 1795 m, 1747 s, 1600 s, 1570 s, 1260 w, 1208 w, 1003 m, 914 s, 861 s, 819 m, 861 s, 819 m, 777 w, 725 s, 689 m.

#### [Yb_2_(mbmp)_3_(thf)_2_] ⋅ 1.5 C_6_D_6_ (4)

Synthesised as per **1** but with ytterbium filings (0.52 g, 3.00 mmol) in place of samarium. Colourless crystals grew overnight from C_6_D_6_ (0.40 g, 27%). M. p. 243–245 °C; *Anal*. Calc. for C_77_H_106_O_8_Yb_2_ (1505.77 g.mol^−1^ after loss of one and a half lattice C_6_D_6_) C 61.42, H 7.10, Yb 22.98. Found: C 61.05, H 6.50, Yb 22.45%. IR (Nujol, cm^−1^): 1943 w, 1738 m, 1569 m, 1259 s, 1120 m, 1093 w, 1023 s, 917 m, 859 s, 798 s, 662 w.

#### [Ce(mbmp)_2_(thf)_2_] ⋅ thf (5)

Synthesised as per **1** but with cerium filings (0.42 g, 3.00 mmol) in place of samarium. Colourless crystals grew after one week from thf (0.05 g, 5%). Owing to the limited yield, no characterisation was obtained other than an X‐ray crystal structure.

#### [Li(thf)_2_La(mbmp)_2_(thf)_2_] (6)

A Schlenk flask equipped with a magnetic stirrer bar was charged with mbmpH_2_ (1.36 g; 4.00 mmol), Hg(C_6_F_5_)_2_ (1.60 g; 3.00 mmol), one drop of Hg metal (to form a reactive lanthanoid‐mercury amalgam) and excess lanthanum filings (0.42 g; 3.00 mmol). Anhydrous thf (∼20 mL) was added by cannula, and the reaction mixture stirred at room temperature for 3 days to form either [La(mbmp)(mbmpH)(thf)_n_] or [La_2_(mbmp)_3_(thf)_x_]. Excess lanthanum metal and mercury were allowed to settle before isolating the supernatant liquid by a filtration cannula. *n*Butylltihium (1.6 M, 0.62 mL, 1.00 mmol) was added to the resulting solution and stirred overnight. The solution was concentrated to ∼5 mL and crystals grew upon standing overnight (0.34 g, 31%). M. p. 228–230 °C; *Anal*. Calc. for C_62_H_92_O_8_LaLi (1111.24 g.mol^−1^): 67.01, H 8.34, La 12.50. Found: C 66.71, H 8.02, La 12.30%. ^1^H‐NMR (400 MHz, C_6_D_6_, 25 °C): δ=7.50 (d, 4H, ArH), 7.19 (d, 4H, ArH), 5.03 (d, 2H, CH_2_), 3.68 (d, 2H, CH_2_), 3.32 (br, 16H, OCH_2_, thf), 2.33 (s, 12H, CH_3_), 1.60 (s, 36H, C(CH_3_)_3_), 1.12 (br, 16H, CH_2_, thf) ppm. IR (Nujol, cm^−1^): 2667 w, 2390 w, 2110 w, 1744 m, 1605 m, 11462 s, 1372 s, 1258 s, 1200 w, 1025 s, 914 m, 861 m, 808 s, 784 m, 730 s.

#### [Li(thf)_2_Pr(mbmp)_2_(thf)_2_] (7)

Synthesised as per **6** but with praseodymium filings (0.42 g, 3.00 mmol) in place of lanthanum to form either [Pr(mbmp)(mbmpH)(thf)_n_] or [Pr_2_(mbmp)_3_(thf)_x_] before treatment with *n*BuLi. Crystals grew overnight from the mother liquor (0.40 g, 36%). M. p. 168–170 °C; *Anal*. Calc. for C_62_H_92_O_8_PrLi (1113.24 g.mol^−1^): C 66.89, H 8.33, Pr 12.66. Found: C 66.17, H 7.69, Pr 12.27%. IR (Nujol, cm^−1^): 2377 w, 2271 w, 2050 m, 1891 m, 1744 s, 1568 s, 1225 s, 918 s, 861 s, 812 m, 722 m, 669 s.

#### [Li(thf)_3_Er(mbmp)_2_] ⋅ 2 C_6_D_6_ (8)

Synthesised as per **6** but with erbium filings (0.50 g, 3.00 mmol) in place of lanthanum to form [Er(mbmp)(mbmpH)(thf)_3_] before treatment with *n*‐BuLi. Crystals grew overnight from C_6_D_6_ (0.53 g, 57%). M. p. 170–172 °C; *Anal*. Calc. for C_50_H_68_O_5_ErLi (923.27 g.mol^−1^ after loss of two coordinated thf and two lattice C_6_D_6_): C 65.04, H 7.42, Er 18.12. Found: C 64.72, H 7.06, Er 17.84%. IR (Nujol, cm^−1^): 2724 s, 2536 w, 2479 w, 2373 w, 2279 w, 2066 m, 1890 m, 1735 s, 1600 s, 1563 m, 1204 w, 1016 m, 922 w, 856 m, 677 m.

#### [Li(thf)_2_Yb(mbmp)_2_(thf)] ⋅ C_6_H_14_ (9)

Synthesised as per **6** but with ytterbium filings (0.52 g, 3.00 mmol) in place of lanthanum to form **4** before treatment with *n*BuLi. A small amount of crystals were grown from a layering of the mother liquor with *n*‐hexane. No further characterisation could be completed.

#### [Li(thf)_2_Lu(mbmp)_2_(thf)] ⋅ 3 C_6_D_6_ (10)

Synthesised as per **6** but with lutetium filings (0.53 g, 3.00 mmol) in place of lanthanum to form [Lu(mbmp)(mbmpH)(thf)_3_] before treatment with *n*‐BuLi. Crystals grew overnight from C_6_D_6_ (0.54 g, 50%). M. p. 230–232 °C; *Anal*. Calc. for C_58_H_84_O_7_LuLi (1075.19 g.mol^−1^ after loss of three lattice C_6_D_6_): C 64.79, H 7.87, Lu 16.27. Found: C 64.28, H 7.35, Lu 15.89%. ^1^H‐NMR (400 MHz, C_6_D_6_, 25 °C): δ=7.34 (br s, 4H, ArH), 7.11 (br s, 4H, ArH), 4.69 (d, 2H, CH_2_), 3.80 (d, 2H, CH_2_), 3.15 (br, 12H, OCH_2_, thf), 2.27 (br s, 12H, CH_3_), 1.60 (s, 36H, C(CH_3_)_3_), 1.05 (br, 12H, CH2, thf) ppm. IR (Nujol, cm^−1^): 2725 w, 2385 w, 2271 m, 1895 w, 1748 s, 1605 s, 1462 s, 1376 s, 1258 m, 1025 m, 861 w, 800 m, 722 m, 673 m.

#### [Li(thf)_4_][Y(mbmp)_2_(thf)_2_]_2_ ⋅ 2 thf (11)

Synthesised as per **6** but with yttrium filings (0.27 g, 3.00 mmol) in place of lanthanum to form [Y(mbmp)(mbmpH)(thf)_3_] before treatment with *n*‐BuLi. Crystals grew overnight from the mother liquor (0.70 g, 66%). M. p. 132–134 °C; *Anal*. Calc. for C_62_H_92_O_8_YLi (1061.24 g.mol^−1^ after loss of two lattice thf and one coordinated thf): C 70.17, H 8.74, Y 8.38. Found: C 69.83, H 8.11, Y 8.05%. IR (Nujol, cm^−1^): 2370 w, 2275 w, 2049 w, 1883 w, 1741 s, 1605 s, 1381 s, 1254 s, 1204 m, 1172 m, 1139 m, 1025 s, 955 w, 865 s, 788 s, 722 m, 677 m.

#### [Li(thf)_4_][Sm(mbmp)_2_(thf)_2_] ⋅ 0.5 thf (12)

Synthesised as per **6** but with samarium filings (0.45 g, 3.00 mmol) in place of lanthanum to form **1** before treatment with *n*BuLi. Crystals grew overnight from the mother liquor (0.54 g, 43%). M. p. 175–177 °C; *Anal*. Calc. for C_70_H_108_O_10_SmLi (1266.90 g.mol^−1^ after loss of half of a lattice thf): C 66.36, H 8.59, Sm 11.87. Found: C 66.12, H 7.95, Sm 11.43%. IR (Nujol, cm^−1^): 2721 m, 2475 w, 2373 m, 2271 m, 2059 m, 1891 s, 1740 s, 1601 s, 1556 s, 1258 w, 1070 w, 874 w, 722 m, 673 s, 583 s.

#### [Li(thf)_4_][Dy(mbmp)_2_(thf)_2_] ⋅ 0.5 thf (13)

Synthesised as per **6** but with dysprosium powder (0.49 g, 3.00 mmol) in place of lanthanum to form [Dy(mbmp)(mbmpH)(thf)_3_] before treatment with *n*‐BuLi. Crystals grew overnight from the mother liquor (0.65 g, 51%). M. p. 200–202 °C; *Anal*. Calc. for C_70_H_108_O_10_DyLi (1279.04 g.mol^−1^ after loss of half of a lattice thf): C 65.73, H 8.51, Dy 12.70. Found: C 65.08, H 7.95, Dy 12.19. IR (Nujol, cm^−1^): 2484 w, 2373 w, 2279 w, 2063 m, 1891 m, 1728 s, 1601 s, 1376 s, 1262 s, 1204 w, 1139 m, 1025 s, 914 m, 861 s, 788 m, 677 m.

#### [Li(thf)_4_][Ho(mbmp)_2_(thf)_2_] ⋅ 0.5 thf (14)

Synthesised as per **6** but with holmium filings (0.50 g, 3.00 mmol) in place of lanthanum to form **3** before treatment with *n*BuLi. Crystals grew overnight from the mother liquor (0.63 g, 63%). M. p. 182–184 °C; *Anal*. Calc. for C_54_H_76_O_6_HoLi (993.05 g.mol^−1^ after loss of four coordinated thf and half of a lattice thf): C 65.31, H 7.71, Ho 16.61. Found: C 64.87, H 7.44, Ho 16.15%. IR (Nujol, cm^−1^): 2725 m, 2586 w, 2365 w, 2275 m, 1907 m, 1732 s, 1601 s, 1556 m, 1204 w, 1143 w, 1074 m, 1029 s, 861 m, 792 m, 722 m, 673 s, 587 s.

#### [AlMe_2_Pr(mbmp)_2_(thf)_2_] ⋅ 2 C_6_D_6_ (15)

Synthesised as per **7** to form either [Pr(mbmp)(mbmpH)(thf)_n_] or [Pr_2_(mbmp)_3_(thf)_x_] then treated with AlMe_3_ (2.00 M, 0.5 mL, 1.00 mmol) in place of *n*‐BuLi. Crystals were grown overnight from C_6_D_6_ (0.32 g, 31%). M. p. 130–132 °C; *Anal*. Calc. for C_56_H_82_O_6_PrAl (1019.14 g.mol^−1^ after loss of two lattice C_6_D_6_): C 66.00, H 8.11, Pr 13.83. Found: C 59.48, H 7.82, Pr 13.51%. IR (Nujol, cm^−1^): 2381 s, 2271 s, 2083 w, 2034 m, 1895 m, 1752 s, 1703 s, 1609 s, 1376 w, 1250 m, 1102 m, 1021 s, 967 w, 865 m, 788 s, 718 m, 692 s.

#### [AlMe_2_Sm(mbmp)_2_(thf)_2_] ⋅ 2 C_6_D_6_ (16)

Synthesised as per **15** but with samarium filings (0.45 g, 3.00 mmol) in place of praseodymium to form **1** before treatment with AlMe_3_. Crystals grew overnight from C_6_D_6_ (0.45 g, 42%). *Anal*. Calc. for C_59_H_82_D_3_O_6_SmAl (1070.66 g.mol^−1^ after loss of one and a half lattice C_6_D_6_): 66.19, H 8.28, Sm 14.04. Found: C 66.04, H 8.13, Sm 13.82%. IR (Nujol, cm^−1^): 2381 s, 2271 s, 2083 w, 2034 m, 1895 m, 1752 s, 1703 s, 1609 s, 1376 w, 1250 m, 1102 m, 1021 s, 967 w, 865 m, 788 s, 718 m, 692 s.

#### [AlMe_2_Tb(mbmp)_2_(thf)_2_] ⋅ 2 C_6_D_6_ (17)

Synthesised as per **15** but with terbium filings (0.47 g, 3.00 mmol) in place of praseodymium to form **2** before treatment with AlMe_3_. Crystals grew overnight from C_6_D_6_ (0.53 g, 51%). M. p. 175–177 °C; *Anal*. Calc. for C_56_H_82_O_6_TbAl (1037.15 g.mol^−1^ after loss of two lattice C_6_D_6_): C 64.85, H 7.97, Tb 15.32. Found: C 64.57, H 7.63, Tb 15.08%. ^1^H‐NMR (400 MHz, C_6_D_6_, 25 °C): δ=7.28 (s, 4H, ArH), 7.18 (s, 4H, ArH), 4.23 (d, 2H, CH_2_), 3.59 (d, 2H, CH_2_ (masked by thf signal), 3.56 (m, 8H, OCH_2_, thf), 2.29 (s, 12H, CH_3_), 1.61 (s, 36H, C(CH_3_)_3_), 0.97 (s, 8H, CH_2_, thf), −0.27 (s, 6H, Al(CH_3_) ppm. IR (Nujol, cm^−1^): 2385 w, 2297 s, 1744 s, 1454 s, 1372 s, 1278 s, 1196 s, 1029 m, 996 s, 914 m, 895 s, 800 m.

#### [La(mbmp)(thf)_4_][AlMe_2_(mbmp)] ⋅ thf (18)

Synthesised as per **15** but with lanthanum filings (0.42 g, 3.00 mmol) in place of praseodymium to form either [La(mbmp)(mbmpH)(thf)_n_] or [La_2_(mbmp)_3_(thf)_x_] before treatment with AlMe_3_. Crystals grew overnight from the mother liquor (0.38 g, 35%). M. p. 180–182 °C; *Anal*. Calc. for C_60_H_90_O_7_LaAl (1089.24 g.mol^−1^ after loss of one lattice and two coordinated thf): C 66.16, H 8.33, La 12.75. Found: C 66.04, H 8.21, La 12.34%. IR (Nujol, cm^−1^): 2373 w, 2283 w, 2030 w, 1891 m, 1744 s, 1605 s, 1239 w, 1021 m, 865 m, 665 m.

#### [K(thf)_3_Gd(mbmp)_2_(thf)_2_] ⋅ 0.5 thf (19)

Synthesised as per **6** but with gadolinium filings (0.47 g, 3.00 mmol) in place of lanthanum to form [Gd(mbmp)(mbmpH)(thf)_3_], and KN(SiMe_3_)_2_ (0.50 M, 0.5 mL, 1.00 mmol) in place of *n‐*BuLi. Crystals grew overnight from the mother liquor (0.65 g, 53%). M. p. 268–270 °C; *Anal*. Calc. for C_66_H_100_O_9_GdK (1233.84 g.mol^−1^ after loss of half of a lattice thf): C 64.25, H 8.17, Gd 12.74. Found: C 63.79, H 7.82, Gd 12.36. IR (Nujol, cm^−1^): 2549 m, 2410 w, 2369 w, 2283 w, 2079 m, 1891 m, 1744 s, 1609 s, 1560 m, 1233 m, 959 w, 681 s, 583 m.

#### [ZnEtYb(mbmp)_2_(thf)] ⋅ 2 C_6_D_6_ (20)

Synthesised as per **6** but with ytterbium filings (0.52 g, 3.00 mmol) in place of lanthanum to form **4**, and ZnEt_2_ (1.00 M, 1.00 mL, 1.00 mmol) was used in place of *n*‐BuLi. Crystals grew overnight from C_6_D_6_ (0.70 g, 66%). M. p. 138–140 °C; *Anal*. Calc. for C_55_H_73_D_3_O_5_YbZn (1058.64 g.mol^−1^ after loss of one and a half lattice C_6_D_6_): C 62.40, H 7.52. Found: C 62.18, H 7.37%. IR (Nujol, cm^−1^): 2553 w, 2434 w, 2377 m, 2267 s, 2132 m, 2034 m, 1748 s, 1605 s, 1376 w, 1208 m, 628 m.

#### Crystal and refinement data

Single crystals covered with viscous hydrocarbon oil were mounted on a glass fibre. Data were obtained at −173 °C (100 K) on the MX1: Macromolecular Crystallography beamline at the Australian Synchrotron, Victoria, Australia. Data collection and integration on the MX1: Macromolecular Crystallography beamline was accomplished using Blu‐Ice.^[27]^ The structures were solved using SHELXS7 and refined by full‐matrix least‐squares on all F2 data using SHELX2014^[28]^ in conjunction with the X‐Seed graphical user interface.^[29]^ All hydrogen atoms were placed in calculated positions using the riding model. Data collection and refinement details are collated in the SI (Table S1).

## Conflict of interest

The authors declare no conflict of interest.

1

## Supporting information

As a service to our authors and readers, this journal provides supporting information supplied by the authors. Such materials are peer reviewed and may be re‐organized for online delivery, but are not copy‐edited or typeset. Technical support issues arising from supporting information (other than missing files) should be addressed to the authors.

Supporting Information

## Data Availability

The data that support the findings of this study are available from the corresponding author upon reasonable request.
