# Supplementary material for: Lanthanoid Biphenolates as a Rich Source of Lanthanoid‐Main Group Heterobimetallic Complexes
Source: Chem Asian J. 2022 Jan 27;17(5):e202101328. doi: 10.1002/asia.202101328 (PMC9303937; doi:10.1002/asia.202101328)
Supplement: Supplementary file 1 — Supporting Information [file ASIA-17-0-s001.pdf]

CHEMISTRY
AN ASIAN JOURNAL

Supporting Information

Lanthanoid Biphenolates as a Rich Source of Lanthanoid-Main Group Heterobimetallic Complexes

Safaa H. Ali, Angus C. G. Shephard, Jun Wang, Zhifang Guo, Murray S. Davies,
Glen B. Deacon, and Peter C. Junk*© 2022 The Authors. Chemistry - An Asian Journal published by Wiley-VCH GmbH. This is an open access article under the terms of the Creative Commons Attribution License, which permits use, distribution and reproduction in any medium, provided the original work is properly cited.

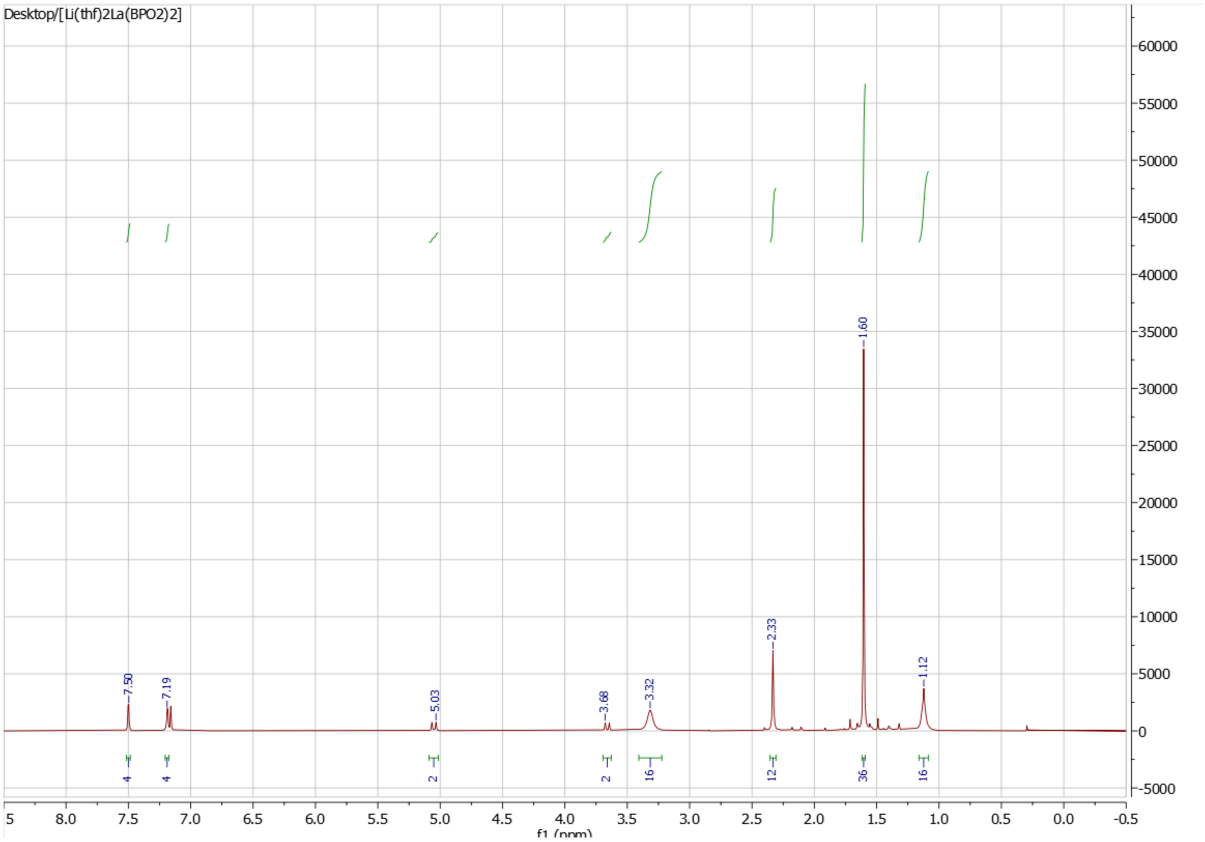

Figure S1 – ^{1}H NMR spectrum of [Li(thf)₂La(mbmp)₂(thf)₂] **(6)**

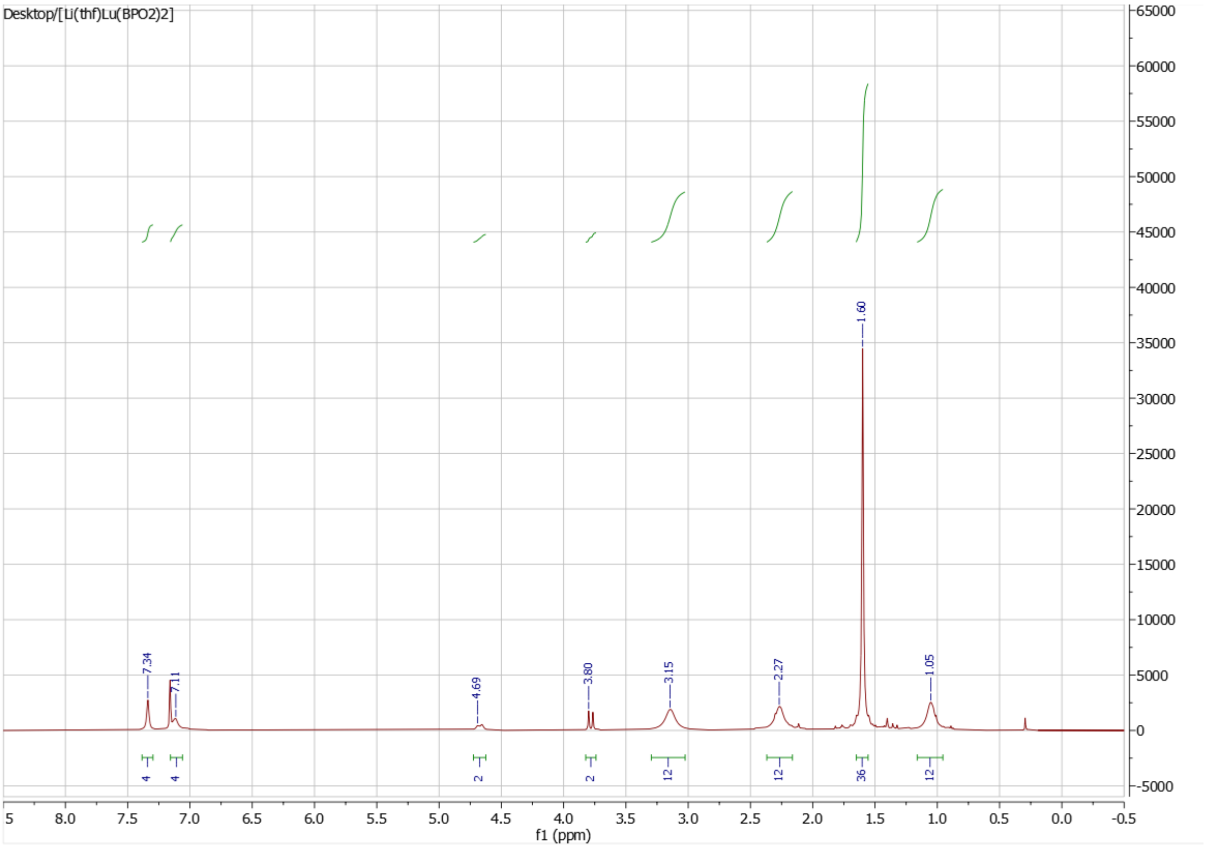

Figure S2 – ^{1}H NMR spectrum of [Li(thf)₂Lu(mbmp)₂(thf)]·3C₆D₆ **(10)**

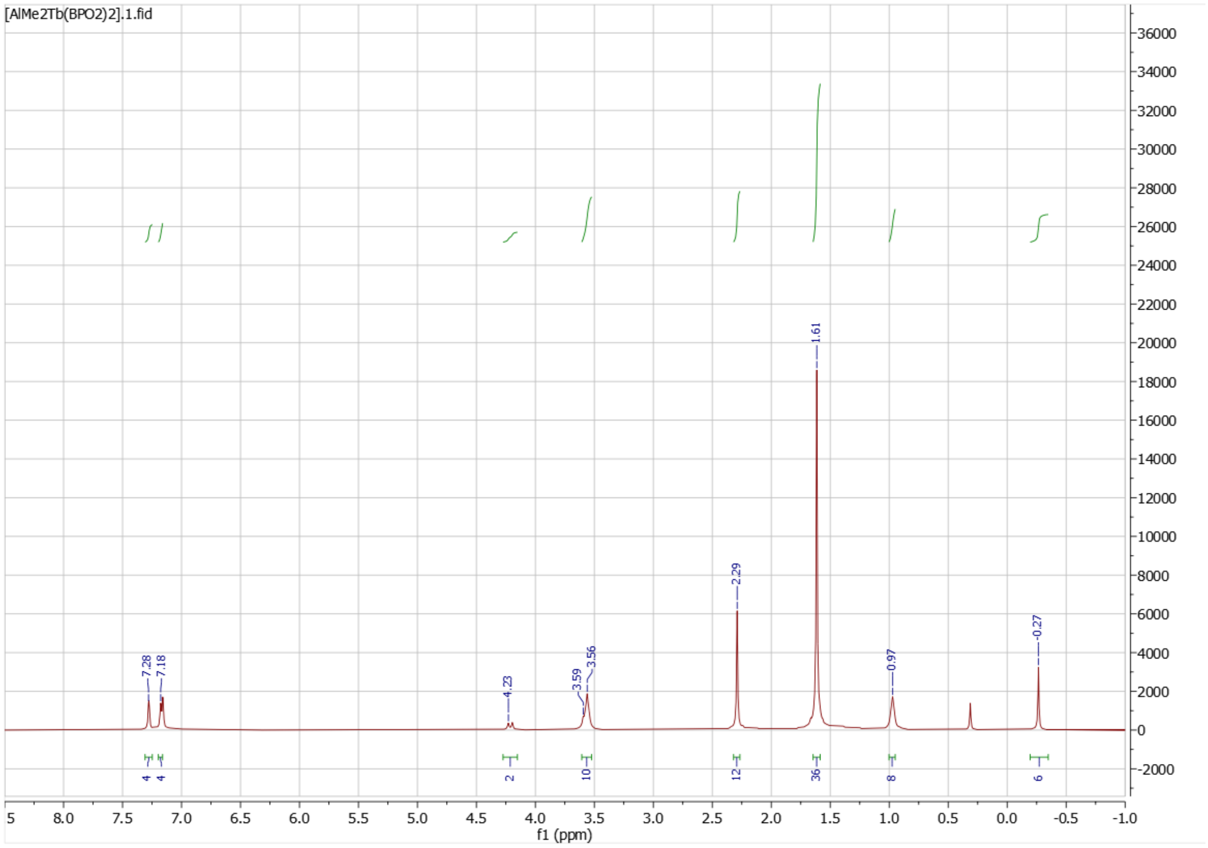

[AlMe2Tb(BPO2)2].1.fid
7.28
7.18
4.23
3.59
3.56
2.29
1.61
0.97
-0.27
4
4
2
10
12
36
8
6
f1 (ppm)

X-ray crystallography:

Single crystals coated with viscous hydrocarbon oil were mounted on glass fibres or loops. Complexes were measured at the Australian Synchrotron on the MX1 beamline, data integration was completed using Blue-ice [1] and XDS [2] software programs. Structural solutions were obtained by either direct methods [3] or charge flipping 4 methods and refined using full-matrix least-squares methods against F^2 using SHELX2018,[5] in conjunction with the X-Seed [6] or Olex2 [6] graphical user interface. All hydrogen atoms were placed in calculated positions using the riding model. Crystal data and refinement details are given in Table S1 and Table S2.

References

[1]. T. M. McPhillips, S. E. McPhillips, H. J. Chiu, A. E. Cohen, A. M. Deacon, P. J. Ellis, E. Garman, A. Gonzalez, N. K. Sauter, R. P. Phizackerley, S. M. Soltis, J. P. Kuhn, J. Synchrotron Radiat. 2002, 9, 401-406.

[2]. W. Kabsch, J. Appl. Crystallogr. 1993, 26, 795.

[3]. G. M. Sheldrick, Acta Crystallogr. Sect. A. 2008, 64, 112-122.

[4]. O. V. Dolomanov, L. J. Bourhis, R. J. Gildea, J. A. K. Howard, H. Puschmann, J. Appl. Crystallogr., 2009, 42, 339-341.

[5]. G. M. Sheldrick, Acta Cryst. 2015, C71, 3-8.

[6]. L. J. Barbour, J. Supramol. Chem., 2001, 1, 189-191.

**Table S1.** Crystal data and structural refinement for lanthanoid complexes **1 - 20**			
	**$[Sm_2(mbmp)_3(thf)_3]\cdot 6thf$ (1)**	**$[Tb_2(mbmp)_3(thf)_3]\cdot 2C_6D_6$ (2)**	**$[Ho_2(mbmp)_3(thf)_2]\cdot 3C_6D_6$ (3)**
Empirical formula	$C_{105}H_{162}O_{15}Sm_2$	$C_{93}H_{126}O_9Tb_2$	$C_{95}H_{124}Ho_2O_8$
Formula weight	1965.04	1705.77	1723.79
Space group	P-1	P-1	$P2_12_12_1$
a/Å	13.264(3)	13.124(3)	17.238(3)
b/Å	16.128(3)	16.719(3)	18.158(4)
c/Å	24.350(5)	21.865(4)	26.717(5)
α/°	84.36(3)	71.03(3)	90
β/°	79.31(3)	75.37(3)	90
γ/°	73.91(3)	68.52(3)	90
Volume/Å^3	4912.0(19)	4173.0(19)	8363(3)
Z	2	2	4
ρ_{calc}g/cm^3	1.329	1.358	1.369
μ/mm^{-1}	1.246	1.737	1.934
Reflections collected	139251	77757	53058
Independent reflections	17285 [R_{int} = 0.0312, R_{sigma} = 0.0141]	14555 [R_{int} = 0.0468, R_{sigma} = 0.0276]	18179 [R_{int} = 0.0418, R_{sigma} = 0.0353]
Data/restraints/parameters	17285/161/1114	14555/28/979	18179/0/959
Goodness-of-fit on F^2	1.25	1.128	1.095
Final R indexes [I>=2σ (I)]	R_1 = 0.0697, wR_2 = 0.1598	R_1 = 0.0296, wR_2 = 0.0667	R_1 = 0.0271, wR_2 = 0.0705
Final R indexes [all data]	R_1 = 0.0736, wR_2 = 0.1623	R_1 = 0.0303, wR_2 = 0.0670	R_1 = 0.0273, wR_2 = 0.0706

	**[Yb_2(mbmp)$_3$(thf)$_2$]·1.5C_6D_6 (4)**	**[Ce(mbmp)$_2$(thf)$_2$]·thf (5)**	**[Li(thf)$_2$La(mbmp)$_2$(thf)$_2$] (6)**
Empirical formula	$C_{86}H_{115}O_8Yb_2$	$C_{58}H_{84}CeO_7$	$C_{62}H_{92}LaLiO_8$
Formula weight	1622.85	1033.37	1111.2
Space group	P-1	$P2_1$	C2/c
a/Å	12.725(3)	11.277	18.145(4)
b/Å	13.175(3)	18.087	17.588(4)
c/Å	23.950(5)	14.159	18.004(4)
α/°	93.97(3)	90	90
β/°	91.56(3)	113.4	98.02(3)
γ/°	92.70(3)	90	90
Volume/Å^3	3999.3(14)	2650.5	5689(2)
Z	2	2	4
ρ_{calc}g/cm^3	1.348	1.295	1.297
μ/mm^{-1}	2.376	0.909	0.804
Reflections collected	25185	44232	26601
Independent reflections	12690 [R_{int} = 0.0301, R_{sigma} = 0.0388]	12145 [R_{int} = 0.0943, R_{sigma} = 0.0788]	4978 [R_{int} = 0.0273, R_{sigma} = 0.0154]
Data/restraints/parameters	12690/202/904	12145/15/612	4978/7/343
Goodness-of-fit on F^2	1.19	1.054	1.121
Final R indexes [I>=2σ (I)]	R_1 = 0.0558, wR_2 = 0.1313	R_1 = 0.0400, wR_2 = 0.0995	R_1 = 0.0265, wR_2 = 0.0644
Final R indexes [all data]	R_1 = 0.0580, wR_2 = 0.1326	R_1 = 0.0410, wR_2 = 0.1001	R_1 = 0.0271, wR_2 = 0.0647

	**[Li(thf)$_2$Pr(mbmp)$_2$(thf)$_2$] (7)**	**[Li(thf)$_3$Er(mbmp)$_2$]·2C$_6$D$_6$ (8)**	**[Li(thf)$_2$Yb(mbmp)$_2$(thf)]·2C$_6$D$_6$ (9)**
Empirical formula	$C_{58}H_{84}LiO_7Pr$	$C_{70}H_{96}ErLiO_7$	$C_{64}H_{98}LiO_7Yb$
Formula weight	1041.1	1223.66	1159.4
Space group	C2/c	P-1	P2$_1$/n
a/Å	18.137(4)	13.704(3)	13.951(3)
b/Å	17.856(4)	15.874(3)	30.577(6)
c/Å	18.039(4)	16.614(3)	14.784(3)
α/°	90	71.89(3)	90
β/°	97.02(3)	76.94(3)	101.09(3)
γ/°	90	69.80(3)	90
Volume/Å^3	5798(2)	3195.2(14)	6189(2)
Z	4	2	4
ρ_{calc}g/cm^3	1.193	1.272	1.244
μ/mm^{-1}	0.886	1.364	1.559
Reflections collected	11095	65025	103503
Independent reflections	4660 [R_{int} = 0.0635, R_{sigma} = 0.0733]	10957 [R_{int} = 0.0378, R_{sigma} = 0.0230]	14752 [R_{int} = 0.0480, R_{sigma} = 0.0248]
Data/restraints/parameters	4660/67/325	10957/0/725	14752/0/676
Goodness-of-fit on F^2	1.019	1.123	1.051
Final R indexes [I>=2σ (I)]	R_1 = 0.0644, wR_2 = 0.1789	R_1 = 0.0245, wR_2 = 0.0585	R_1 = 0.0336, wR_2 = 0.0853
Final R indexes [all data]	R_1 = 0.0901, wR_2 = 0.2003	R_1 = 0.0246, wR_2 = 0.0586	R_1 = 0.0394, wR_2 = 0.0881

	**$[Li(thf)_2Lu(mbmp)_2(thf)]\cdot3C_6D_6$ (10)**	**$[Li(thf)_4]_2[Y(mbmp)_2(thf)_2]_2\cdot2thf$ (11)**	**$[Li(thf)_4][Sm(mbmp)_2(thf)_2]\cdot0.5thf$ (12)**
Empirical formula	$C_{76}H_{102}LiLuO_7$	$C_{148}H_{232}Li_2O_{22}Y_2$	$C_{72}H_{112}LiO_{10.5}Sm$
Formula weight	1309.48	2555.02	1302.9
Space group	P-1	P-1	$P2_1/n$
a/Å	12.698(3)	14.142(3)	12.984(3)
b/Å	14.857(3)	17.118(3)	37.320(8)
c/Å	19.178(4)	30.888(6)	15.010(3)
α/°	79.95(3)	105.00(3)	90
β/°	81.34(3)	92.42(3)	106.79(3)
γ/°	80.65(3)	90.60(3)	90
Volume/$Å^3$	3487.2(13)	7214(3)	6963(3)
Z	2	2	4
$\rho_{calc}g/cm^3$	1.247	1.176	1.243
μ/mm^{-1}	1.466	0.864	0.899
Reflections collected	65057	184689	69146
Independent reflections	12236 [R_{int} = 0.0339, R_{sigma} = 0.0208]	25428 [R_{int} = 0.0480, R_{sigma} = 0.0230]	12144 [R_{int} = 0.0514, R_{sigma} = 0.0317]
Data/restraints/parameters	12236/14/791	25428/248/1653	12144/44/826
Goodness-of-fit on F^2	1.076	1.127	1.102
Final R indexes [I>=2σ (I)]	R_1 = 0.0257, wR_2 = 0.0643	R_1 = 0.0895, wR_2 = 0.2429	R_1 = 0.0331, wR_2 = 0.0799
Final R indexes [all data]	R_1 = 0.0264, wR_2 = 0.0647	R_1 = 0.0968, wR_2 = 0.2482	R_1 = 0.0335, wR_2 = 0.0802

	$[Li(thf)_4][Dy(mbmp)_2(thf)_2]\cdot 0.5thf$ (13)	$[Li(thf)_4][Dy(mbmp)_2(thf)_2]\cdot 0.5thf$ (14)	$[AlMe_2Pr(mbmp)_2(thf)_2]\cdot 2C_6D_6$ (15)
Empirical formula	$C_{72}H_{112}DyLiO_{10.5}$	$C_{72}H_{112}HoLiO_{10.5}$	$C_{68}H_{94}AlO_6Pr$
Formula weight	1315.05	1317.48	1175.32
Space group	$P2_1/n$	$P2_1/n$	$P2_1/n$
a/Å	13.015(3)	12.997(3)	10.0098(10)
b/Å	37.279(8)	37.232(8)	19.875(2)
c/Å	14.908(3)	14.912(3)	32.781(3)
α/°	90	90	90
β/°	106.40(3)	106.40(3)	94.664(4)
γ/°	90	90	90
Volume/$Å^3$	6939(3)	6922(3)	6500.1(11)
Z	4	4	4
$\rho_{calc}g/cm^3$	1.259	1.264	1.201
μ/mm^{-1}	1.132	1.199	0.81
Reflections collected	83826	64325	54468
Independent reflections	12074 [R_{int} = 0.0707, R_{sigma} = 0.0398]	12023 [R_{int} = 0.0579, R_{sigma} = 0.0360]	11381 [R_{int} = 0.0843, R_{sigma} = 0.0639]
Data/restraints/parameters	12074/44/826	12023/44/826	11381/7/670
Goodness-of-fit on F^2	1.153	1.139	1.086
Final R indexes [I>=2σ (I)]	R_1 = 0.0387, wR_2 = 0.0905	R_1 = 0.0347, wR_2 = 0.0815	R_1 = 0.0697, wR_2 = 0.1790
Final R indexes [all data]	R_1 = 0.0396, wR_2 = 0.0909	R_1 = 0.0357, wR_2 = 0.0820	R_1 = 0.0909, wR_2 = 0.2000

	$[AlMe_2Sm(mbmp)_2(thf)_2]\cdot 2C_6D_6$ (16)	$[AlMe_2Tb(mbmp)_2(thf)_2]\cdot 2C_6D_6$ (17)	$[AlMe_2(mbmp)][La(mbmp)(thf)_4]\cdot thf$ (18)
Empirical formula	$C_{68}H_{94}AlO_6Sm$	$C_{68}H_{94}AlO_6Tb$	$C_{72}H_{114}AlLaO_{10}$
Formula weight	1184.76	1193.33	1305.52
Space group	$P2_1/n$	$P2_1/n$	C2/c
a/Å	9.8600(10)	10.0041(10)	17.306(4)
b/Å	19.597(2)	19.930(2)	20.132(4)
c/Å	32.532(3)	32.763(3)	39.877(8)
α/°	90	90	90
β/°	94.696(4)	94.621(4)	94.87(3)
γ/°	90	90	90
Volume/$Å^3$	6264.9(11)	6511.1(11)	13843(5)
Z	4	4	8
$\rho_{calc}g/cm^3$	1.256	1.217	1.253
μ/mm^{-1}	1	1.147	0.685
Reflections collected	47053	68539	88121
Independent reflections	10452 [R_{int} = 0.0465, R_{sigma} = 0.0323]	11455 [R_{int} = 0.2448, R_{sigma} = 0.2274]	16538 [R_{int} = 0.0396, R_{sigma} = 0.0247]
Data/restraints/parameters	10452/0/679	11455/7/670	16538/21/776
Goodness-of-fit on F^2	1.062	0.962	1.127
Final R indexes [I>=2σ (I)]	R_1 = 0.0520, wR_2 = 0.1329	R_1 = 0.0814, wR_2 = 0.1993	R_1 = 0.0451, wR_2 = 0.1063
Final R indexes [all data]	R_1 = 0.0535, wR_2 = 0.1343	R_1 = 0.1996, wR_2 = 0.2639	R_1 = 0.0508, wR_2 = 0.1092

	$[K(thf)_3Gd(mbmp)_2(thf)_2]\cdot 0.5thf$ (19)	$[ZnEtYb(mbmp)_2(thf)]\cdot 2C_6D_6$ (20)
Empirical formula	$C_{68}H_{104}GdKO_{9.5}$	$C_{64}H_{85}O_5YbZn$
Formula weight	1269.86	1172.72
Space group	$P2_1/c$	Cc
a/Å	15.065(3)	23.231(5)
b/Å	17.317(4)	13.716(3)
c/Å	28.544(6)	19.869(4)
α/°	90	90
β/°	95.09(3)	106.51(3)
γ/°	90	90
Volume/Å^3	7417(3)	6070(2)
Z	4	4
ρ_{calc}g/cm^3	1.137	1.283
μ/mm^{-1}	0.998	1.971
Reflections collected	103361	24428
Independent reflections	13082 [R_{int} = 0.0947, R_{sigma} = 0.0526]	9186 [R_{int} = 0.0570, R_{sigma} = 0.0600]
Data/restraints/parameters	13082/455/891	9186/60/634
Goodness-of-fit on F^2	1.088	1.014
Final R indexes [I>=2σ (I)]	R_1 = 0.0472, wR_2 = 0.1213	R_1 = 0.0335, wR_2 = 0.0674
Final R indexes [all data]	R_1 = 0.0870, wR_2 = 0.1471	R_1 = 0.0443, wR_2 = 0.0712
